# Elucidation of Factors Affecting the Age-Dependent Cancer Occurrence Rates

**DOI:** 10.3390/ijms26010275

**Published:** 2024-12-31

**Authors:** Jun Xiao, Yangkun Cao, Xuan Li, Long Xu, Zhihang Wang, Zhenyu Huang, Xuechen Mu, Yinwei Qu, Ying Xu

**Affiliations:** 1 College of Computer Science and Technology, Jilin University, Changchun 130012, China; xiaojun20@mails.jlu.edu.cn (J.X.); xuanli0518@gmail.com (X.L.); zhihang22@mails.jlu.edu.cn (Z.W.); zhenyuh19@mails.jlu.edu.cn (Z.H.); 2 Systems Biology Laboratory for Metabolic Reprogramming, School of Medicine, Southern University of Science and Technology, Shenzhen 518055, China; caoyk20@mails.jlu.edu.cn (Y.C.); xul6@sustech.edu.cn (L.X.); m250921296@gmail.com (X.M.); 3 School of Artificial Intelligence, Jilin University, Changchun 130012, China; 4 School of Mathematics, Jilin University, Changchun 130012, China

**Keywords:** cancer occurrence, Fenton reaction, growth signal, cell cycle, viral infection

## Abstract

Cancer occurrence rates exhibit diverse age-related patterns, and understanding them may shed new and important light on the drivers of cancer evolution. This study systematically analyzes the age-dependent occurrence rates of 23 carcinoma types, focusing on their age-dependent distribution patterns, the determinants of peak occurrence ages, and the significant difference between the two genders. According to the SEER reports, these cancer types have two types of age-dependent occurrence rate (ADOR) distributions, with most having a unimodal distribution and a few having a bimodal distribution. Our modeling analyses have revealed that (1) the first type can be naturally and simply explained using two age-dependent parameters: the total number of stem cell divisions in an organ from birth to the current age and the availability levels of bloodborne growth factors specifically needed by the cancer (sub)type, and (2) for the second type, the first peak is due to viral infection, while the second peak can be explained as in (1) for each cancer type. Further analyses indicate that (i) the iron level in an organ makes the difference between the male and female cancer occurrence rates, and (ii) the levels of sex hormones are the key determinants in the onset age of multiple cancer types. This analysis deepens our understanding of the dynamics of cancer evolution shared by diverse cancer types and provides new insights that are useful for cancer prevention and therapeutic strategies, thereby addressing critical gaps in the current paradigm of oncological research.

## 1. Introduction

The systematic examination of the age-dependent occurrence rates for different cancer types given in the SEER reports [1,2] revealed that (1) the vast majority of the cancer types each have an age-dependent unimodal distribution for the occurrence rate, namely the occurrence rate going up with age until it reaches a peak age and then going down with age, and (2) the few remaining types each have a bimodal distribution with the additional peak due to viral infection (see Results). Excluding the contribution by viral infection, all the cancer types in TCGA [3] each have a unimodal distribution among adult patients. The main question we address in this study is as follows: why does each cancer type have a unimodal distribution of age-dependent occurrence rate?

Numerous studies have been published regarding cancer occurrence rates. For example, multiple researchers attribute the increase in cancer occurrence with age to the accumulation of mutations in our genomes as we age [4,5,6], while the age-dependent decline in immune function—and hence the reduced ability to detect and destroy cancer cells—is considered another significant factor contributing to the age-dependent increase in cancer occurrence rate [7,8,9]. Moreover, age-associated, chronic, low-grade inflammation has been linked to a progressive increase in cancer incidence [10,11]. These should lead to the conclusion that cancer occurrence rates go up with age, but in reality, cancer occurrence rates decline with age once beyond the occurrence rate peak age(s) in general. A recent study suggested that the expression of the NUPR1 protein increases with age, which affects iron metabolism and induces a state of iron deficiency in cells, thereby limiting the ability of cancer cells to proliferate [12,13]. 

While these studies offer explanations to some of the age-dependent behaviors in cancer occurrence for some cancers, they lack a general and quantitative understanding of the universal behaviors across all cancer types, which has limited the possible applications of the studies.

In this study, we focus on the unimodality of the age-dependent cancer occurrence rate distributions across a range of cancer types. Our previous study statistically demonstrated that two distinct factors can explain the unimodality of such distributions for three cancer types, namely testicular cancer, triple-negative breast cancer, and cervical cancer [14]: (i) the age-dependent cancer risk level, defined as the average number of stem cell divisions (SCDs) in the relevant organ from birth to a specific age, which increases with age [15], and (ii) the age-dependent availability of growth signals, specifically needed by a cancer (sub)type, which generally decreases with age. Supplementary Figure S1 shows a schematic illustration of the idea.

Our study here expands on our previous work [15] to more cancer types with more rigorous analyses and offers more insights about the possible roles played by growth signals throughout cancer development, consisting of the following new elements:

1. Roles played by growth signals in cancer cell cycle progression;
2. Hints about the cancer cell cycle program, which is clearly different from the normal cell cycle program of human cells;
3. Roles played by viral infection in cancer development;
4. Gender disparity for the vast majority of cancer types;
5. Roles played by sex hormones in the development of cancer types having early peak ages in the occurrence rate distributions.

Overall, by analyzing the age-dependent occurrence rate (ADOR) distributions, we have gained a few new insights about cancer development, which have been largely ignored by the cancer research community or mostly fragmented to say the least. Some of these new insights could provide novel and useful guidance for developing fundamentally novel ways to treat cancer.

## 2. Results

### 2.1. The Landscape of Age-Dependent Occurrence Rates of 23 Cancer Types

We examined the age-dependent occurrence rates of 23 cancer types in the TCGA database [3], representing all cancer types that meet the following criteria: (1) the occurrence rates for the cancer type are available in the SEER database [1,2], and (2) the cancer is a solid tumor. The correspondence between SEER cancer sites and TCGA cancer types is detailed in Table 1. This section summarizes the characteristics of the ADOR distributions across the 23 cancer types.

**Figure 1:**
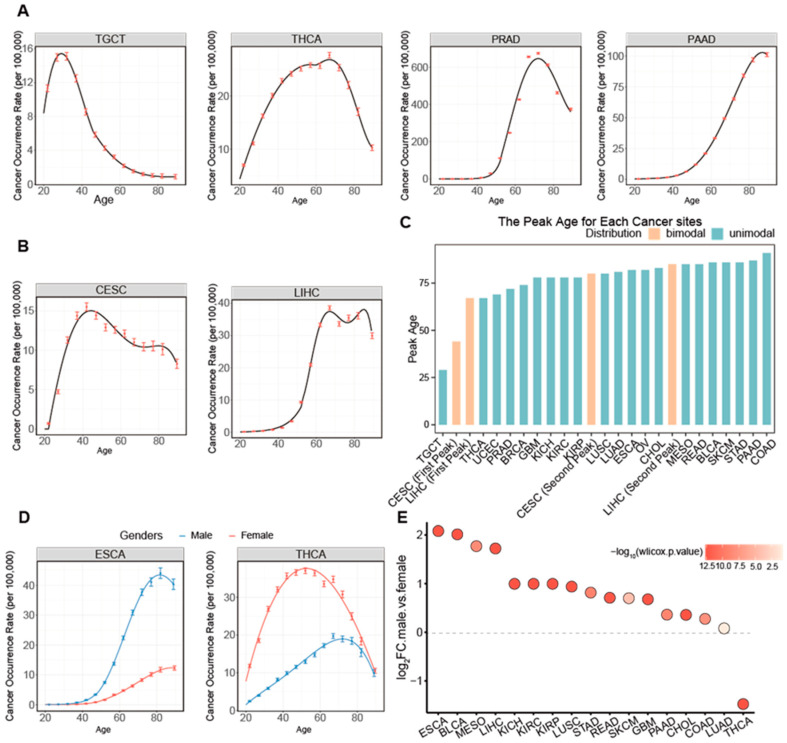
The landscape of age-dependent occurrence rates of different cancers. (**A**,**B**) Fitting for occurrence rate distributions having unimodality (**A**) and bimodality (**B**) with 95% confidence intervals being depicted by the red lines. (**C**) The peak ages of cancer occurrence rate distributions across different cancer types. (**D**) Comparison between cancer ADOR distributions of female and male patients of ESCA and THCA. (**E**) Fold changes in cancer occurrence rates in male vs. female.

Of the 23 cancer types, 21 have a unimodal distribution, as depicted in Figure 1A and Figure S2, while 2 cancer types, cervical and liver cancers, exhibit a bimodal distribution, as shown in Figure 1B and Supplementary Table S1. Figure 1C shows that different cancer types exhibit distinct peak ages, ranging from the youngest at age 29 for testicular cancer to the oldest at 90 for colon cancer.

Of the 23 cancer types that can occur in both genders, 17 exhibit significant differences in ADOR distributions between the two genders (Figure 1D and Figure S3). Among these, 16 have higher occurrence rates in males, with the disparity ranging from 1.06- to 4.23-time of those in females. Thyroid cancer is the only exception, for which females have a 2.78-fold occurrence rate compared to that in males (Figure 1E).

The primary objective of this study is to address key questions derived from these observations about the ADOR distributions: (1) Why do a vast majority cancer types each exhibit a unimodal ADOR distribution? (2) Why do cervical and liver cancers each display a bimodal distribution? (3) What accounts for the significant differences in cancer occurrence rates between males and females for the 17 cancer types? (4) What are the principal determinants of the distinct peak ages observed among the 21 cancer types?

### 2.2. Cancers with Unimodal ADOR Distributions

Our previous study has demonstrated that each of the unimodal ADOR distributions are statistically attributed to two main factors: the total number of SCDs in the host organ from birth to the age of cancer diagnosis, termed the risk level, and the level of availability of the growth signals in blood circulation specifically required by the cancer, called the support level [14]. Building on this discovery, we conducted a focused analysis on 7 of the 21 cancer types: COAD, ESCA, LUAD, PAAD, SKCM, TGCT, and THCA, representing all the cancer types with the age-dependent risk level data available from Tomasetti et al. [15].

For the risk level of each cancer, we used the total number of SCDs in the host organ from birth to the age of cancer diagnosis (Table S2 and Method 6). For the support level of each cancer, we identified growth signals, growth factors, and/or sex hormones specific for each cancer type, as defined in the Methods (Table S3, and Method 7).

For each of the seven cancer types, we successfully built an accurate regression model for their ADOR distributions against the age-dependent distribution of the risk level and the age-dependent distributions of the support levels, by using the all-subsets regression analyses. Details follow for each of the seven cancers.

Model for ESCA: Let function $f_{1}\left(x\right)$ be the age-dependent distribution of cancer risk level (Table S2). By applying the method outlined in Method 7, we have predicted *BMP6*, *TGFα*, and *TGFβ1* as the growth signals specifically needed by the cancer type. $f_{2}\left(x\right)$, $f_{3}\left(x\right)$, and $f_{4}\left(x\right)$ are their age-dependent distributions, respectively, with x being the age (Table S3). Detailed information is given in Tables S4–S6. Let $y\left(x\right)$ be the ADOR distribution of the cancer, as reported in the SEER database (Table S1); $y^{p}\left(x\right)$ is a continuous function that approximates $y\left(x\right),$ derived through a regression analysis (Table S7). The following is our regression model of the ADOR distribution against the cancer risk and cancer support levels:

(1) $$y^{p}\left(x\right)=-176.42*f_{1}\left(x\right)-1.38*f_{2}\left(x\right)-8.89*f_{1}\left(x\right)*f_{3}\left(x\right)+8*10^{-1}*f_{1}\left(x\right)*f_{4}\left(x\right)+345.93$$

**Figure 2:**
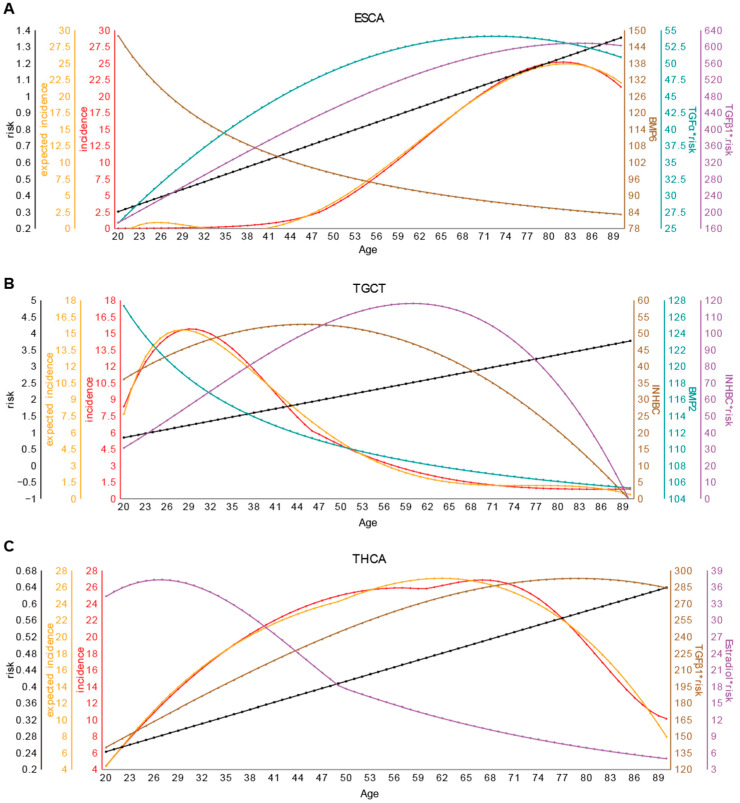
Regression models of age-dependent (x-axis) cancer occurrence rates against cancer risk and the availability levels of growth signals needed by each cancer type. (**A**–**C**) The regression models for (**A**) ESCA, (**B**) TGCT, and (**C**) THCA, respectively. The red line represents the cancer occurrence rate; the orange line is the predicted occurrence rate based on cancer risk level (black line), and the other lines are for the concentrations of circulatory growth signals. The symbol * represents the interaction term, indicating the product of cancer risk and the concentration of circulatory growth signals.

This model has an accuracy level, $y^{p}\left(x\right)$ vs. $y\left(x\right),$ at $R_{adjust}^{2}=0.9979$ with *p*-value < $10^{-87}$. The regression model and the actual distribution are illustrated in Figure 2A.

Published data strongly support our prediction of *BMP6*, *TGFα*, and *TGFβ1* being growth signals used by ESCA. The expression of *BMP6* correlates with cancer progression and prognosis [16,17], and *BMP6* is known to promote invasion and metastasis [17]. Published studies have shown that the repression of *ACVR1*, *BMP6*’*s* receptor, reduces cell growth [17]. *EGFR*, the receptor of *TGFα*, is highly expressed in ESCA, which is known to be associated with the poor prognosis of a cancer [18,19,20]. Furthermore, ESCA patients tend to exhibit elevated serum levels of *TGFβ1* compared to healthy controls, which generally goes down following radiotherapy, highlighting its relevance to cancer progression [21]. It has also been reported that the upregulation of *TGFβ*, along with the increased expression of *VEGF*, is linked to larger tumor sizes in ESCA [22,23].

Model for TGCT: Here, $y\left(x\right)$, $y^{p}\left(x\right)$, and $f_{1}\left(x\right)$ are defined similarly to those defined in the previous subsection. *INHBC* and *BMP2* are identified as the growth signals specifically needed by the cancer type, and $f_{2}\left(x\right)$ and $f_{3}\left(x\right)$ are their age-dependent distributions, respectively. Detailed information is given in Tables S1–S7. The following is our regression model of the cancer’s ADOR distribution against cancer risk and caner support levels:

(2) $$y^{p}\left(x\right)=-63.07*f_{1}\left(x\right)-2.13*f_{2}\left(x\right)-4.97*f_{3}\left(x\right)+3.28*10^{-1}*f_{1}\left(x\right)*f_{2}\left(x\right)+761.66$$

This model has an accuracy level at $R_{adjust}^{2}=0.9956$ with a *p*-value < $10^{-76}$. The regression model and the actual distribution are displayed in Figure 2B.

Published data support our prediction of *INHBC* and *BMP2* being growth signals used by TGCT. The inhibin/activin signaling pathway is known to play a significant role in the development and progression of TGCT, of which inhibin-βC (*INHBC*) is a subunit [24]. Similarly, *BMP2* has been found to promote cancer growth, invasion, and migration across a variety of cancer types [25,26,27,28].

Model for THCA: $y\left(x\right)$, $y^{p}\left(x\right)$, and $f_{1}\left(x\right)$ are defined similarly to those defined above. *TGFβ1* and estradiol (*E2*) are identified as the growth signals specifically needed by the cancer type (see Method 7), and $f_{2}\left(x\right)$ and $f_{3}\left(x\right)$ are their age-dependent distributions, respectively. Detailed information is given in Tables S1–S7. The following gives the model for the ADOR distribution of THCA against cancer risk and cancer support levels:

(3) $$y^{p}\left(x\right)=-130.87*f_{1}\left(x\right)+4.42*10^{-1}*f_{1}\left(x\right)*f_{2}\left(x\right)+2.86*10^{-1}*f_{1}\left(x\right)*f_{3}\left(x\right)-35.63$$

The model has an accuracy level at $R_{adjust}^{2}=0.9901$ with a *p*-value < $10^{-66}$. The regression model and the actual distribution are displayed in Figure 2C.

*TGFβ1* is known to enhance cell proliferation and invasion in THCA through upregulating the expression of *HMGA1* via the PI3K/Akt signaling pathway [29,30]. *E2* is also known to drive the proliferation and growth of THCA [31]. These findings support our model.

The gender disparity in the occurrence rates of THCA, with women having over two times the rate of cancer occurrence by men, strongly suggesting a significant role for estrogen in this cancer’s development (Figure 1D,E). This is further supported by the fact that estrogen-related genes and genes co-expressed with *E2* are known to play critical roles in the progression of THCA [32]. These findings collectively provide strong support to our model.

Similar analyses are conducted on LUAD, SKCM, PAAD, and COAD cancers with comparable results, supports, and conclusions, as detailed in Table S7 and Figure S4. Table 2 summarizes the key results for each of the seven cancer types.

Overall, the analysis results of the seven cancer types here demonstrated the efficacy of our approach in using age-dependent cancer risks and supports to accurately predict cancer occurrence rates. The accuracy levels of the regression models further support our overall hypothesis: it takes two factors for a cancer to take place, one being the internal reason for the affected cells to continuously proliferate for survival, based on our model [40], and another being the growth signals specifically needed by the cancer type.

### 2.3. Roles Played by Growth Signals in Cancer Cell Proliferation

The above analyses have revealed that each cancer (sub)type typically requires 1 to 3 growth signals to accurately explain its ADOR distribution. Here, we study how these growth signals may contribute to the cell cycle progression of a cancer. It is noteworthy that a fundamental difference between cancerous cell division and a normal one is that the normal cell division is triggered and coordinated by developmental and/or tissue repair signals in a top-down manner, while cancerous cell division is a bottom-up process, driven by the continuous biosynthesis of nucleotides induced to keep the intracellular pH stable [40,41,42]. To demonstrate the bottom-up nature of a cancerous cell cycle process, we have conducted a co-expression analysis of the core cell cycle genes (see Methods) involved in the human cell cycle program in each of the seven cancer types studied in the above section, with the detailed gene list given in Table S4.

Figure S6 show the heatmap of the co-expressed cell cycle genes in each of the seven cancer types. From the figure, we can see that the cell cycle program consists of a few co-expressed gene clusters for each cancer type. Our hypothesis here is as follows: each cell cycle gene cluster is driven independently by at least one growth signal or by the de novo synthesis of nucleotides, which is induced to adapt to the stressor of persistent intracellular alkalization [40,41,42]. It is noteworthy that growth signals play necessary roles for a cancer to take place for at least some cancer types, such as estrogen-dependent breast cancers [43] and androgen-dependent prostate cancers [44,45], for which cancer cells will die if the needed growth signals are not available [46]. In addition, our previous work has demonstrated that the de novo synthesis of deoxyribonucleotides is a major driver for the cell cycle program in cancer [42]. Here, we determine which specific cell cycle clusters may be driven by DNA/nucleotide synthesis.

**Figure 3:**
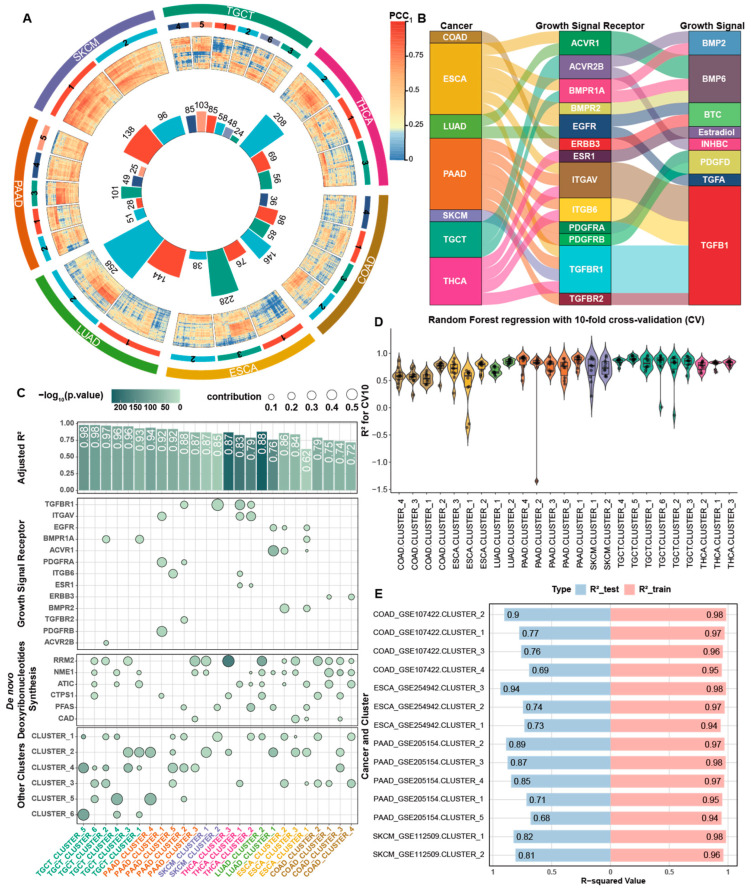
Co-expression patterns of cell cycle genes. (**A**) Circos plot illustrating the co-expression patterns of cell cycle genes in seven cancer types. The outermost circle lists cancer types, followed by a middle circle representing co-expression clusters of cell cycle genes, and an inner circle with a heatmap detailing gene correlations (row and column orders are identical). At the center, the bar plot shows the gene numbers for each co-expression clusters. The colors for cancer types are consistent with those used in other panels of this figure. (**B**) The Sankey diagram showing the predicted growth signals and their receptors for regression model of ADOR in the seven cancer types. (**C**) Combination chart presenting regression analysis for PC1 of each co-expressed cluster against growth signal-related receptors, genes involved in de novo deoxyribonucleotides synthesis, and PC1 of other cell cycle cluster(s). The top bar chart shows adjusted R^2^ values, with color indicating *p*-values. The lower bubble chart depicts the contribution of each factor to the regression model, with color coding for *p*-values. (**D**) Violin plots showing the R^2^ values for random forest-based regressions with 10-fold cross-validation (CV). (**E**) Bar plots for the R^2^ values for random forest-based regressions in both the training and test sets using the independent datasets.

To demonstrate the above hypothesis computationally, we have conducted a co-expression analysis among the cell cycle genes in each of the seven cancer types, using consensus clustering with the same parameters (Method 9). This results in two to six co-expressed clusters of cell cycle genes, with each consisting of 24 to 258 genes (Figure 3A) across the seven cancer types, with the detailed gene list for each cluster of each cancer type given in Table S8.

We have then demonstrated that, for each of the seven cancer types, the PC1 of each cluster of the cancer type can be well explained by the receptors of the predicted growth factors for the cancer type (Figure 3B), de novo synthesis of deoxyribonucleotides, and/or PC1 of the other clusters of the cancer. Regression analyses revealed that these factors collectively contribute to cell cycle progression (Figure 3C and Table S8).

To validate the robustness of our predictions, we applied a random forest-based regression to fit the PC1 of each cluster for each cancer type against the selected features in both our primary datasets and four independent datasets. Higher accuracy on both the training and test sets suggests the effectiveness of these factors in explaining cell cycle progression across different cancer types (Figure 3D,E).

### 2.4. Cancers with Bimodal Occurrence Rate Distributions

To elucidate the factors that contribute to the bimodal ADOR distributions of liver and cervical cancers (Figure 1B), we have considered the impact of viral infection, as our preliminary study strongly suggests that the first peak is due to such infection and conducted analyses on the second peak in a similar manner to those having unimodal distributions.

**Figure 4:**
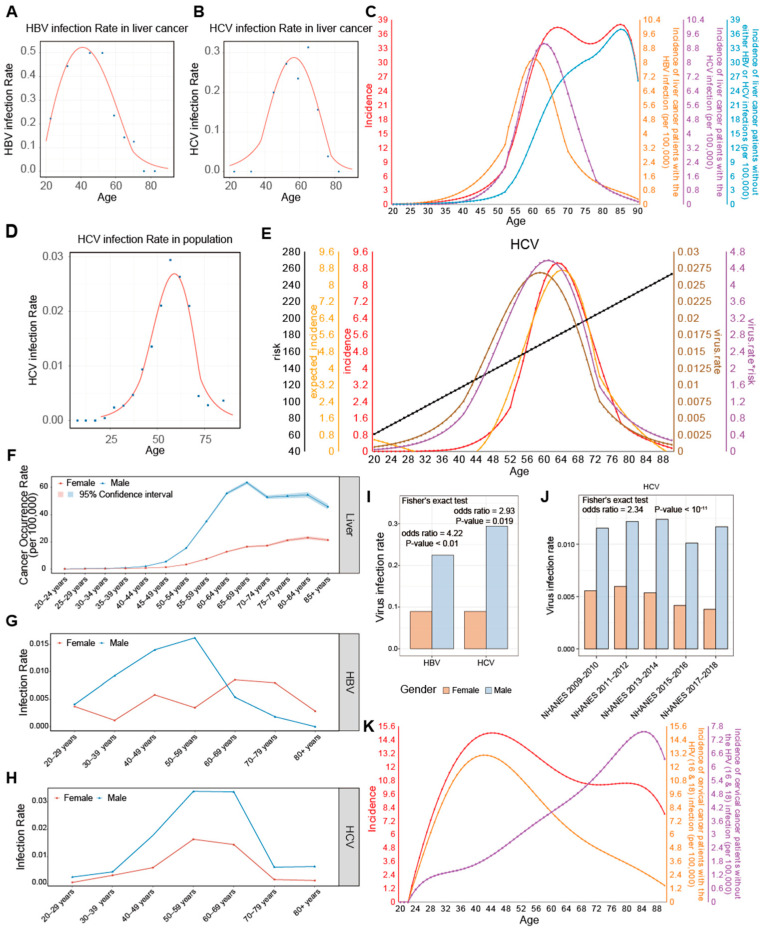
Viral infection rates related to cancer occurrence rate. (**A**,**B**) Regression models of HBV (**A**) and HCV (**B**) infection rates in liver cancer. (**C**) Liver cancer occurrence rates with HBV infection, with HCV infection, and without viral infection. (**D**) The regression model for age-dependent HCV infection rate in the USA population. (**E**) The regression model for cancer occurrence rates of patients with HCV infection against cancer risk and viral infection rate in the USA population. (**F**) Differences in age-dependent occurrence rates of liver cancer between female and male. (**G**,**H**) Examination of gender differences in age-dependent viral infection rates in the U.S. population for HBV (**G**) and HCV (**H**). (**I**) The HBV and HCV infection rates in liver cancer patients by gender. (**J**) HCV infection rate among population, categorized by gender and shown across different NHANES datasets. (**K**) Cervical cancer occurrence rates with HPV 16/18 infection and those without such infection. The symbol * represents the interaction term, indicating the product of cancer risk and the concentration of circulatory growth signals.

Liver cancers with HBV/HCV infection: Hepatitis B virus (HBV) and hepatitis C virus (HCV) are risk factors for liver cancer [47,48]. We analyzed liver cancer occurrence rates among patients with and without such infections. Consider the following two functions:

(4) $$y_{v}\left(x\right)=y\left(x\right)*f_{c}^{v}\left(x\right)$$

(5) $$y_{nv}\left(x\right)=y\left(x\right)*(1-f_{c}^{v}\left(x\right))$$

where $y\left(x\right)$ is the ADOR distribution of liver cancer derived from the SEER data (Figure 1B), and $f_{c}^{v}\left(x\right)$ is the age-dependent conditional probability of viral infection among liver cancer patients, based on data from TCGA (Figure 4A,B and Figure S7A and Table S9).

We have then examined the ADOR distributions for liver cancers with HBV and HCV infections, separately. We note that they each exhibit a unimodal distribution, with both peaks closely aligned with the first peak of the bimodal occurrence rate distributions of the liver cancer (Figure 4C). We then analyzed the ADOR distribution of liver cancer without viral infections and noted that this distribution is basically unimodal, with its peak closely aligned with the second peak of the bimodal distribution (Figure 4C).

Based on these observations, we have conducted regression analyses for the unimodal occurrence rate distributions of liver cancers, with and without viral infections ($y^{p}\left(x\right)$), separately. As before, we first constructed the following distributions: (1) the age-dependent distributions of the infection rates of HBV and HCV, respectively, (2) the age-dependent risk level distribution of liver cancer, and (3) the age-dependent distributions of support levels, based on the identified growth signals specifically needed by liver cancer with and without viral infection, where age-dependent infection rates of HBV and HCV are collected from population-based viral infection data in the NHANES database (Figure 4D and Figure S7B, Table S9).

For liver cancers with HCV infection, we have constructed the following regression model:

(6) $$y^{p}\left(x\right)=-1.03*10^{-2}*f_{1}\left(x\right)-940.06*f_{2}\left(x\right)+7.24*f_{1}\left(x\right)*f_{2}\left(x\right)+1.54$$

where x denotes age, $f_{1}\left(x\right)$ is the age-dependent distribution of cancer risk, and $f_{2}\left(x\right)$ is a continuous function approximating the age-dependent HCV infection rate in the USA. The model has an accuracy level at $R_{adjust}^{2}=0.9829$ with a *p*-value < $10^{-58}$, as shown in Figure 4E. Similarly, for liver cancer with HBV infection, we have the following regression model:

(7) $$y^{p}\left(x\right)=1.21*f_{1}\left(x\right)-4.87*10^{-3}*f_{2}\left(x\right)+34.37*f_{1}\left(x\right)*f_{2}\left(x\right)+5.15*10^{-1}*f_{3}\left(x\right)-2.21*10^{-3}*f_{1}\left(x\right)*f_{3}\left(x\right)-289.11$$

where $f_{1}\left(x\right)$ is the age-dependent distribution of risks for liver cancer, $f_{2}\left(x\right)$ is the age-dependent distribution of HBV infection rates, and $f_{3}\left(x\right)$ is the age-dependent distribution of the blood level of *TGFβ1*. The model has an accuracy level at $R_{adjust}^{2}=0.8524$ with a *p*-value < $10^{-25}$. The detail is depicted in Figure S7C.

For liver cancer without viral infection, we developed a regression model for the unimodal distribution of the occurrence rates of liver cancers ($y^{p}\left(x\right)$) as follows:

(8) $$y^{p}\left(x\right)=-35.6*f_{1}\left(x\right)-7.68*f_{2}\left(x\right)+1.3*10^{-2}*f_{1}\left(x\right)*f_{2}\left(x\right)+1.38*10^{-1}*f_{1}\left(x\right)*f_{3}\left(x\right)-3.21*10^{3}$$

where $f_{1}\left(x\right)$ is the age-dependent distribution of cancer risk, $f_{2}\left(x\right)$ and $f_{3}\left(x\right)$ are regression models for the age-dependent available levels of the identified growth signals *TGFβ1* and *PDGFD*, specifically needed by liver cancer, respectively. The model has an accuracy level at $R_{adjust}^{2}=0.9883$ with a *p*-value < $10^{-63}$, as illustrated in Figure S7D.

*PDGFD* has been implicated in promoting angiogenesis, proliferation, and metastasis in liver cancer [49,50]. It is noteworthy that the bimodal occurrence rate distribution of liver cancer is only observed in male patients, while a unimodal distribution is observed among female patients, according to the SEER reports (Figure 4F). To understand the difference between the two populations, we note that the age distribution of the viral infection rate for females in the USA is more spread out compared to that for males who have a peak at age 50–69 (Figure 4G–H), which gives rise to the additional peak in the liver cancer occurrence rate distribution. Further, the infection rates for HBV and HCV are consistently higher in males than in females, both in liver cancer patients and the general US population (Figure 4I,J and Figure S7E). These observations strongly suggest that viral infections are a key factor contributing to the gender-specific differences in the occurrence rate distribution of liver cancer.

Cervical cancers with HPV infection: Among the 200 types of human papillomavirus (HPV) identified, HPV 16 and 18 are known to strongly associate with the increased risk of several cancers, including cervical, vaginal, anal, penile, and oropharyngeal cancers [51]. They account for ~70% of cervical cancer cases globally (Figure S8A) [52]. We have observed that cervical cancer patients infected with HPV 16 or 18 were significantly younger than those infected with other HPV types or without HPV infection (Figure S8B). This observation underscores the critical role of these HPV types in the development of early-onset cervical cancer. In the following, HPV infection refers to infection by HPV 16 or 18 (Figure S8C).

Similar to our analyses on liver cancer, we consider the cervical cancer cases with HPV infection and without, separately. Let $y_{H}\left(x\right)$ denote the occurrence rate for cervical cancer with HPV infection and $y_{nH}\left(x\right)$ the occurrence rate for cervical cancer without HPV infection, with their ADOR distributions shown in Figure 4K and detailed in Table S9. Notably, the peak age for $y_{H}\left(x\right)$ is 42 years old, coinciding with the first peak of the overall bimodal distribution ($y\left(x\right)$), and the peak age for $y_{nH}\left(x\right)$ is 85.

Regression analyses for the unimodal distributions for the two cases were performed separately. Considering the relatively low number of samples, we used the log2(fold change) > 0 as the cutoff for differentially expressed genes in this analysis. For cervical cancers with HPV infection, we have constructed the following regression model:

(9) $$y^{p}\left(x\right)=-249.42*f_{1}\left(x\right)-102.73*f_{2}\left(x\right)-2.66*f_{3}\left(x\right)+2.54*f_{1}\left(x\right)*f_{3}\left(x\right)+207.09$$

where $f_{1}\left(x\right)$ is the age-dependent distribution of cancer risk, $f_{2}\left(x\right)$ is a continuous function approximating the age-dependent HPV infection rate in the USA, and $f_{3}\left(x\right)$ is the age-dependent distribution of blood level of *TGFα*, identified to be the sole growth signal specifically needed for this class of cervical cancer. Since the Tomasetti et al. paper [15] did not include cervical cancer, hence no SCD data, we used the lifetime risk from the SEER reports to represent the cancer risk, which is known to have a significant correlation with SCD [15]. The model has an accuracy level at $R_{adjust}^{2}=0.9971$ with a *p*-value < $10^{-82}$, as shown in Figure S8D.

For cervical cancers without HPV infection, we have constructed the following regression model:

(10) $$y^{p}\left(x\right)=17.56*f_{1}\left(x\right)-1.36*10^{-3}*f_{2}\left(x\right)-1.6*10^{-2}*f_{1}\left(x\right)*f_{2}\left(x\right)+1.34$$

where $f_{1}\left(x\right)$ is the age-dependent distribution of the cancer risk, and $f_{2}\left(x\right)$ is the age-dependent distribution of blood level of *TGFβ1*, identified to be the sole growth signal specifically needed for this class of cervical cancer. The model has an accuracy level at $R_{adjust}^{2}=0.9752$ with a *p*-value < $10^{-53}$, as shown in Figure S8E.

This comprehensive analysis underscores the pivotal role of viral infections in defining the occurrence rate patterns of liver and cervical cancers, with distinct epidemiological impacts evident in our statistical models and corroborated by extensive data analysis.

### 2.5. Understanding Gender Disparity in Cancer Occurrence Rates

To understand why cancer occurrence rates are consistently higher in males than in females for a majority, specifically 16 of the 17 cancer types, as depicted in Figure 1E, we analyzed differentially expressed genes (DEGs) across 10 relevant types of normal tissues in the GTEx database [53], each having at least 10 samples for both genders (Table 3 and Method 12). We identified between 24 and 405 protein coding genes upregulated in males and between 26 and 359 of such genes in females across these tissue types (Figure S9A). Notably, a significant portion of these genes are located on sex chromosomes—between 5.6% and 23.5% of the upregulated genes in females in the X chromosome and between 3.7% and 58.3% of the upregulated genes in males in the Y chromosome. Additionally, substantial differences were also observed in genes not located on sex chromosomes, detailed in Figure S9B,C.

**Figure 5:**
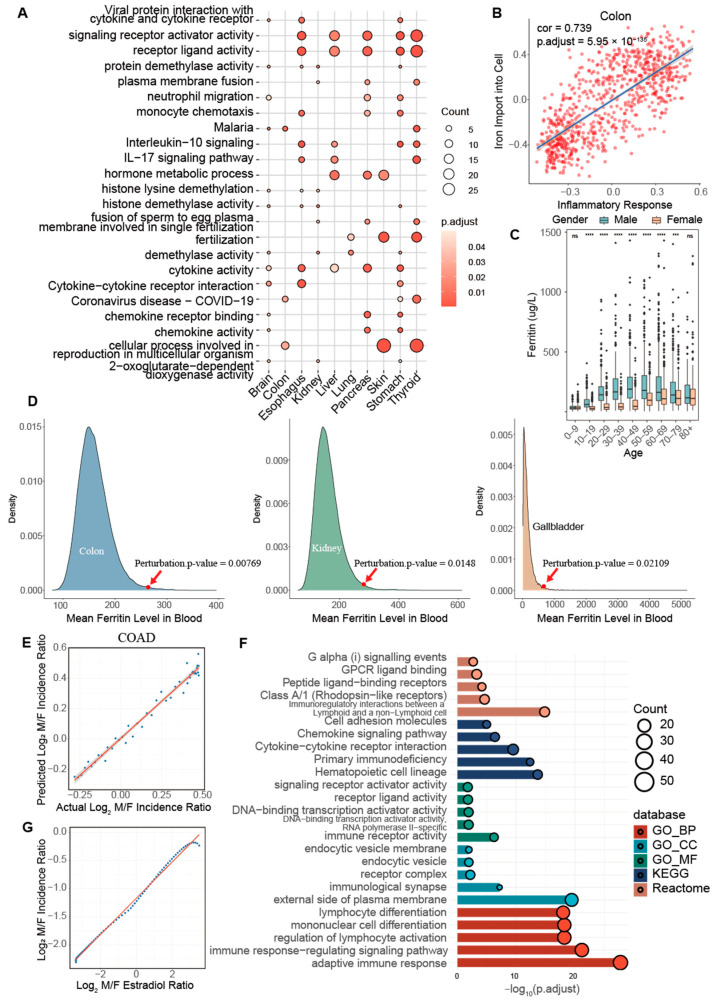
Gender- and organ-specific disparity in cancer occurrence rates. (**A**) Enriched pathways among upregulated genes in males vs. females across different organs, focused on pathways enriched in at least three organ types. (**B**) Correlation between immune responses and iron levels in colon cancer, illustrating a significant association. (**C**) Box plots for variations in ferritin levels between genders across different age groups, measured using the Wilcoxon test for statistical significance. Significance levels are indicated as ‘ns’ for not significant, ‘***’ for *p*-value ≤ 0.001, and ‘****’ for *p*-value ≤ 0.0001. (**D**) Levels of blood ferritin levels across different cancer types, where 100,000 random reassignments of cancer and non-cancer labels among samples. (**E**) Scatter plots comparing the actual cancer incidence ratio (male vs. female) in different ages with those predicted by the expression difference of iron-related genes for COAD. The blue dots represent the actual (x-axis) and predicted (y-axis) ratios for specific ages, with the red line showing a linear regression, indicating strong agreement between the values. (**F**) Enriched pathways among upregulated genes in female thyroid tissues, highlighting the most enriched pathways in different databases. (**G**) Scatter plots showing the regression analysis for cancer incidence ratio (male vs. female) against fold change of *E2* (male vs. female) in THCA. Each blue dot represents a specific age point, with the red line indicating the linear regression, demonstrating that the estradiol ratio effectively explains the incidence ratio in THCA.

Enrichment analyses of the DEGs revealed multiple biological functions that differ between the genders. Upregulated genes in males were significantly enriched in cytokine activity, interleukin signaling, monocyte chemotaxis, and neutrophil migration, underscoring potential mechanisms that contribute to the gender disparity in cancer occurrence (Figure 5A). Cytokine signaling and chemokine activities are central to chronic inflammation [54,55]. IL-17, a key pro-inflammatory cytokine [56], enhances the production of various cytokines and chemokines for recruiting neutrophils and monocytes [57,58]. The continuous presence and activities of the innate immune cells can lead to prolonged inflammation and subsequent tissue damages [59], contributing to the higher inflammatory levels in male cancer sites.

Inflammation is recognized as a hallmark of cancer, facilitating and possibly driving tumor progression and sustainability [60,61]. One contributing factor to chronic inflammation is persistent iron accumulation [62], which can catalyze the formation of the hydroxyl radical through the Fenton reaction:

(11) $${Fe}^{2+}+H_{2}O_{2}\to {Fe}^{3+}+\cdot OH+{OH}^{-}$$

The hydroxyl radical can damage cellular components, such as lipids, proteins, and DNA, and exacerbate inflammation [63,64]. Notably, the iron level has a significant and positive correlation with oxidative stress and inflammation across multiple tissues (Figure 5B, Figures S10 and S11). Higher serum levels of iron, ferritin, transferrin, and hemoglobin are observed in males compared to females (Figure 5C and Figure S9D–F). In addition, a perturbation analysis (Method 14) linking cancer labels and the ferritin concentration detected a positive association with higher serum ferritin levels in several cancer types (Figure 5D). This association is particularly pronounced in conditions such as hereditary hemochromatosis, where excessive iron accumulation significantly elevates cancer risk, including in colon, rectal, prostate, and breast cancers [65].

Our regression analysis has revealed that the expression difference of iron-related genes can account for the variations in age-dependent cancer occurrence rates between genders across multiple cancer types (Figure 5E and Figure S12), with the $R_{adjust}^{2}$ value ranging from 0.9529 to 0.9695 (Table S10). This evidence suggests that iron accumulation may substantially heighten the risk of cancer by fueling the Fenton reactions, particularly in males.

Compared to these cancer types, thyroid cancer is a notable exception with a higher occurrence rate in females than males. This difference is reflected by the significant enrichment of inflammation-related pathways by upregulated genes in female thyroid tissues (Figure 5F), which potentially increase the risk of thyroid cancer. Additionally, women are more susceptible to autoimmune thyroid disorders, such as Hashimoto’s thyroiditis and Graves’ disease, which are linked to chronic inflammation [66,67]. Furthermore, our analysis has investigated the role of *E2* in thyroid cancer occurrence. A regression analysis indicates that varying the *E2* level in blood can explain the difference in age-dependent cancer occurrence rates between genders in thyroid cancer with $R_{adjust}^{2}$ being 0.996 (Figure 5G and Table S10).

This comprehensive analysis underscores the critical role of iron accumulation and inflammation in mediating gender-based differences in cancer occurrence rates. The *E2* level might be a key reason for the difference between the occurrence rates of thyroid cancer in men and women. It offers valuable insights into the underlying biological mechanisms that could inform targeted therapeutic strategies and preventive measures. By understanding these gender-specific pathways, we can more effectively tailor interventions to manage or mitigate the risk factors associated with iron dysregulation, inflammation, and sex hormones.

### 2.6. Role of Sex Hormones in Cancers with the Early Cancer Occurrence Rate Peaks

**Figure 6:**
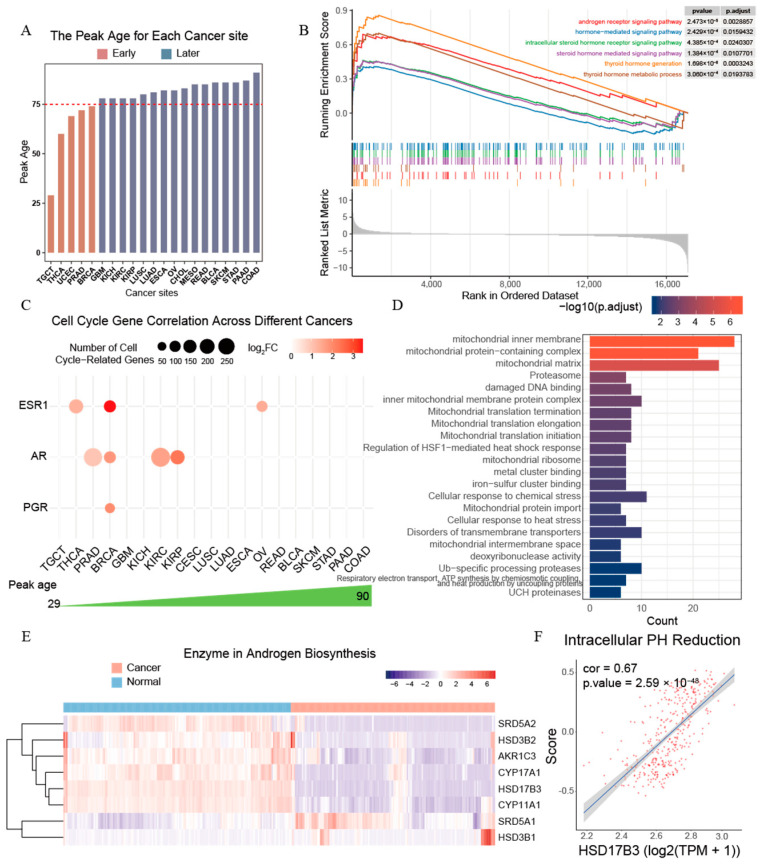
Cancers with early (age < 75) vs. late (≥75) peak ages. (**A**) Cancer types arranged in the increasing peak age with early ones colored in brown and late ones in blue. (**B**) Gene set enrichment analysis (GSEA) results in cancers with early peak ages vs. those in cancers with late peak ages. (**C**) Sex hormones identified to be cell cycle driving across different cancers. (**D**) Pathway enrichment analysis among genes that show a negative correlation with *AR* expression in TGCT. (**E**) Heatmap displaying the differential expression of enzyme genes involved in androgen biosynthesis between normal tissues and cancerous tissues. (**F**) Correlations between *HSD17B3* expression and intracellular pH reduction signals in normal testicular tissue.

To understand the differences in the peak ages of cancer occurrence rate distributions across different cancer types, we categorized all 21 cancer types with unimodal ADOR distributions into two groups based on their peak ages: early (age < 75) and late (age > 75) (Figure 6A). Compared to cancers with late peak ages, the enrichment analyses of differentially expressed genes reveal that cancers with early peak ages exhibit significantly higher activities related to sex hormones and their receptors (Table S11, Figure 6B, and Method 16). This finding suggests that sex hormones may play a crucial role in the early onset of certain cancers.

Sex hormones, including androgens, estrogens, and progestogens, generally decrease with age and have been implicated in the pathogenesis of multiple cancer types [68,69]. Notably, major sex hormone receptors significantly enhance cancer growth in cancers that present at early peak ages (Figure 6C, Table S6). These cancers often originate in gender-specific organs that rely on sex hormones for growth. For example, estrogen and progesterone are known to be associated with certain subtypes of breast cancers [43,70], and the hormonal imbalances during post-menopause are major risk factors for corpus uteri cancer [71]. Androgens are known to be involved in the development of prostate cancer [44,45]. Similarly, thyroid cancer, which also exhibits an early peak age, is influenced by estrogen [31].

Testicular cancer, having the earliest peak age, at age 29, among all cancers studied here, seems to be more complex, as androgens do not seem to be directly involved in testicular cancer development [72], evidenced by the significant downregulation of the androgen receptor (*AR*) and *HSD17B3*, an enzyme critical for testosterone biosynthesis in testicular cancer compared to normal testicular tissues (Figure S13A–C).

To investigate the reason for the inhibition of testosterone and its receptor *AR* in the development of testicular cancer, we identified genes whose expressions correlate negatively with *AR* (PCC < −0.3 and FDR < 0.05). Pathway enrichment analyses of these genes reveal that iron–sulfur clusters accumulate in mitochondria and damage mitochondrial proteins, which may inhibit *AR* signaling (Figure 6D). Additionally, key enzymes involved in androgen biosynthesis are significantly downregulated in testicular cancer (Figure 6E). The analyses of the androgen biosynthesis pathway reveal that the synthesis of testosterone consumes six H^+^ (Figures S14 and S15). Our previous study has shown that cancer tissue cells tend to repress alkalizing reactions, since their intracellular space is being persistently alkalized by Fenton reactions [41,42]. We also observed that intracellular pH reduction positively correlates with the biosynthesis of testosterone, as seen in the enzyme *HSD17B3* and *AR* levels in normal testis, suggesting their sensitivity to pH changes (Figure 6F and Figure S13D). In addition, genes involved in the estrogen biosynthesis pathway, which is closely related to androgen biosynthesis, are also markedly suppressed (Figures S13E and S16). Based on these, we predict that it is the persistent Fenton reactions in mitochondria that result in the reduced activities of the androgen biosynthesis pathway in testicular cancer.

## 3. Discussion

We presented a computational study aiming to provide an explanation of the observed patterns of age-dependent occurrence rates across numerous solid tumor cancers. Our explanation is simple and supported by the published literature. Namely, the unimodular distributions are the result of two main factors: the age-dependent number of stem cell divisions in the relevant organ since birth on average [15] and the age-dependent availability level of bloodborne growth signals specifically required by a cancer type [43,44,45,70,73]. The bimodular distributions of a few cancer types are the result of two contributors, one being age-related, as in the case of unimodular distributions, and the other being the result of viral infection [74,75].

There have been published studies trying to explain the age-dependent nature of cancer occurrence rates, as discussed in the Introduction section [4,5,6,7,8,9,10,11,12,13]. The main difference between our study and those studies lies in that (1) our explanation is systematic and quantitative, applicable to all cancer types, and (2) our study provides additional new insights about cancer evolution, which are driven by internal reasons, ultimately due to chronic inflammation and iron accumulation [76,77], and enabled by external factors, namely the availability levels of bloodborne growth signals specifically needed by a cancer type.

The published cancer studies tend to focus on the internal reasons of why a cancer develops in a tissue, due to chronic inflammation, possibly as the result of persistent exposure to carcinogens or genetic mutations. Our study here provided strong evidence that, for a cancer to take place, it also requires external help, namely growth signals in circulation. Two types of data strongly support our new framework: (1) the roles of sex hormones have been systematically studied for a few gender-specific cancer types, such as hormone-sensitive breast cancer and prostate cancer, leading to the conclusion that eliminating the availability of the relevant sex hormones slowdown or even stop the development of such cancers [43,44,45,70]; and (2) multiple cancer types have associated atrophic lesions, including atrophies associated with stomach cancer [78], prostate cancer [79,80], breast cancer [81,82], testicular cancer [83], liver cancer [84], pancreatic cancer [85], and brain cancer [86,87], which we predict are the result of a lack in growth signals specifically needed by a cancer type as outlined below.

Based on our model of cancer development, persistent cell division in cancer is driven by the continuous de novo biosynthesis of nucleotides induced for rapid H^+^ production to keep the intracellular pH stable in cells affected by intracellular Fenton reactions [40,42]:

(12) $$H_{2}O_{2}+O_{2}^{∙-}\overset{{Fe}^{2+}}{\to }{OH}^{-}+\cdot OH+O_{2}$$

Specifically, persistent Fenton reactions, fueled by the continuous production of $H_{2}O_{2}$ and $O_{2}^{∙-}$ by inflammatory neutrophils and macrophages [41], continuously produce ${OH}^{-}$ and alkalizing the intracellular space, casting a major stressor and killing the affected cells if ${OH}^{-}s$ are not neutralized in a timely manner. Simple solutions like utilizing H^+^ pumps or exchangers do not solve the issue, as they will either violate the cell’s electroneutrality or disrupt other ions’ homeostasis [88], which leads to the affected cells activating a range of acidifying reprogrammed metabolisms to keep the intracellular pH stable [42]. Among them, nucleotide de novo biosynthesis and sialic acid synthesis and deployment are the predominant acidifiers across all the cancer types in TCGA [42], with the former driving cell division [41,42].

While the level of the intracellular Fenton reaction dictates the rate of cell proliferation, so the synthesized nucleotides can be released extracellularly in a sustained and timely manner [41,42], it takes growth signals to make this happen, as demonstrated in this study. Figure S17 strongly suggests that the affected cells will die in the form of tissue atrophy. This model explains why various cancers tend to have atrophy associated with the development of the cancer. This strongly points to a new direction for cancer treatment, namely via cleaning up the relevant growth signals in circulation.

While this study provides valuable insights into the role of growth signals in cancer development, several issues might limit the wide applications of our analysis tools. The first is that the growth signals are currently predicted based on all the samples of individual cancer types, each of which generally can be further partitioned into subtypes. Samples of each subtype might require fewer growth signals than predicted in this study. Another limitation lies in the functional studies of detailed roles played by each growth signal as well as the level of contribution by each growth signal in pushing the whole cell cycle program moving in a coherent manner, while knowing such information may provide more informative guidance to design drugs that can bind with a target growth signal to prevent the signal from binding to its receptor, hence possibly disrupting the cell cycle program and killing the cancer cell.

## 4. Materials and Methods

### 4.1. Data Collection

RNA-seq data for this study were systematically retrieved from the XENA platform at the University of California, Santa Cruz (UCSC) [89], which integrates multiple publicly available cancer datasets, including the TCGA [3] and GTEx databases [53]. Our analysis here focused on transcriptomic data from cancerous tissues in TCGA and normal tissues from GTEx [89,90]. For breast cancer analyses, only female patients were used.

Data were downloaded using the platform’s data portal, with samples selected based on tissue type and disease state. The preliminary data processing steps, including quality control, normalization, and batch effect correction, were performed using the Bioconductor package DESeq2 [91] in R (version 4.2.3), ensuring comparability across different datasets. Only the protein coding genes were considered in this work.

### 4.2. Identification of Differentially Expressed Genes (DEGs)

For each cancer type, DEGs were identified by comparing the raw count data of gene-expression levels between cancer tissues from the TCGA and matching control tissues from the GTEx using the DESeq2 package (version 1.38.3), which tests for differential expression using a model based on the negative binomial distribution. A gene was considered as a DEG if it has fold change > 1.5 and FDR < 0.001.

### 4.3. Enrichment Analyses

The pathway and functional enrichment analyses were conducted among our interesting gene sets, using the ClusterProfiler package (version 4.6.3) [92] against the following databases: GO Biological Processes, KEGG Pathways, and Reactome Pathways. Statistical significance was determined using the hypergeometric test. Pathways were considered enriched if the FDR < 0.05.

### 4.4. Calculation of Gene Set Scores Using GSVA

To assess the activity of a specific pathway across selected samples and calculate the gene set scores, we performed a log2 transformation (log2(TPM + 1)) on the gene expression data represented in TPMs, referred to as log2TPM. We then applied gene set variation analysis (GSVA) to calculate gene set scores [93]. Information about the gene sets used, including their sources, is listed in Table S12.

### 4.5. Regression Models for Age-Dependent Cancer Occurrence Rates

Occurrence rate data for the cancer types considered in this study from 2015 to 2019 were obtained from the SEER database, accessed using SEER*Stat software (version 8.4.3) [2]. These data were mapped to the corresponding cancer (sub)types in TCGA as listed in Table 1, assisted with a classification scheme based on information including cancer sites and histological characteristics of cancers to ensure correct matches between datasets.

Considering that the data given in the SEER reports are cancer occurrence rates at a few discrete ages, typically at 20, 30, …, 80, we have used a continuous function to approximate these data points to facilitate our modeling work. This is achieved through a regression analysis. Specifically, for a given set of age–cancer occurrence rate pairs and the analytical form of a function, a linear regression was performed to model their relationship, using the *lm* function in the R package to optimally determine the parameter values of the function. This approach enabled the tailoring of regression models to the specific characteristics of the age-related patterns observed in the data, ensuring a precise understanding of how occurrence rates vary with age across different cancer types.

We carried this out for each cancer type based on the available set of age–cancer occurrence rate pairs. All regression coefficients in each model were statistically significant, with *p*-values < 0.05 (Table S1).

### 4.6. Estimating the Age-Dependent Cancer Risk Level in an Organ

The total number of stem cell divisions (SCDs) in each organ was collected from the data provided by Tomasetti et al. [15], which consisted of the (average) number of stem cells (s) in a fully developed organ and the (average) number of divisions per stem cell in the organ each year ($d_{yr}$), as detailed in Table S2. To estimate the (average) age-specific number of stem cell divisions (SCDs) in an organ, we applied the formula given in the work of Tomasetti et al. [15]:

(13) $$SCD\left(age\right)=\sum_{n=1}^{{log}_{2}S}2^{n}+s*d(age)$$

where $d(age)=age*d_{yr}$ represents the total number of per stem cell divisions from birth to the specified age, and s is the (average) number of stem cells in a fully developed organ. The first part of the formula represents the stem cell divisions starting from the first precursor cell to generate a fully developed tissue, and the latter part represents the further division, due to normal tissue turnover, until the specified age of that tissue. It is noteworthy that the first part of the formula is approximately equal to 2 s^−2^.

Due to the potentially large values of SCD(age), ranging from 10^8^ to 10^12^, we scaled these numbers down by a factor of 10^9^ for computational ease in our analyses.

### 4.7. Estimating the Age-Dependent Levels of Growth Signals Needed by a Cancer (Sub)Type

We employed the following procedure to estimate the (average) age-dependent levels of growth signals in the blood circulation needed by a specified cancer (sub)type:

(1) Identification of core cell cycle genes: We manually curated a list of 1608 cell cycle genes collected from the literature [94] (Table S4). Recognizing that some cell cycle genes may be involved in other biological processes, and thus, their expression levels might not directly reflect the activity level of the cell cycle, we conducted an analysis to iteratively filter out those that are not strongly co-expressed with at least N distinct core cell cycle genes across all the samples of the target cancer type, resulting in a list of core cell cycle genes.

Specifically, we used the following parameters in this analysis: two genes are considered as co-expressed if the Pearson correlation coefficient (PCC) between their expression profiles is >0.3 with FDR < 0.05; and N is set as 10. In addition, we ranked all the core cell cycle genes based on the number of their strongly co-expressed genes and filtered out the bottom third of the core cell cycle genes with the fewest co-expressed genes.

(2) Prediction of cell cycle driving hormones/growth signals: We have collected all bloodborne peptide hormones and growth factors from the UniProt database [95] through keyword searches: KW-0372 for hormones and KW-0339 for growth factors [96]. We have also collected receptors for the identified hormones and growth signals via keyword (KW-0675) searches in UniProt, coupled with searches in CellPhoneDB and CellTalkDB [97,98], which contain the pairing information between hormone/growth signal and receptors (Table S5).

We predict that a hormone/growth factor is a candidate driving signal for cell cycle program if its receptor has the mean TPM value > 5 across all cancer samples; it is at least 1.5-fold upregulated in cancer vs. the matching control samples; and its expression profile across the cancer samples is co-expressed with at least 10% of the core cell cycle genes, namely PCC > 0.3 with FDR < 0.05 (Table S6).

(3) Estimation of age-dependent level of a bloodborne hormone/growth signal: For each predicted hormone/growth factor, we collected the following information from the Human Protein Atlas (HPA) [99]: (1) the major organs that synthesize the hormone/growth signal; and (2) whether the signal can be secreted into the blood circulation. If the signal is indeed bloodborne, we estimate its age-dependent level as follows:

For each such signal g, let X be the set of organs that produce g, and $L_{x}(g,age)$ be the average expression level of g across all samples at a given age for each organ x∈X collected from GTEx [90]. To estimate the age-dependent production level of g by all relevant organs, we use the following formula:

(14) $$\sum_{x\in X}{\omega_{x}L}_{x}\left(g,age\right)$$

where $\omega_{x}$ is a scaling factor that reflects the level of contribution by organ x to g’s total production, determined as follows: $\omega_{x}$ = 1.0 if x is a major organ, and $\omega_{x}$ = 0.5 if x a minor organ for producing g. If the organ producing g is located in the brain, excluding the hypothalamus, we will exclude it from our analyses, because the blood–brain barrier prevents such hormones from crossing out of the brain. Additionally, the age-dependent levels of non-protein hormones in circulation were indirectly collected from NHANES [100].

We then constructed a continuous function through a linear regression analysis as in Section 4.5 in Methods, which optimally fits the production levels given by $\sum_{x\in X}{\omega_{x}L}_{x}(g,age)$ across different ages. Across all g considered in this study, our predicted regression model for each achieved R^2^ > 0.6 with a *p*-value < 0.05 (Table S3).

### 4.8. Regression Analyses for Cancer Occurrence Rate Against Cancer Risk and Growth Signals

For each cancer type considered in this study, a linear regression analysis was conducted for the ADOR distribution against the age-dependent cancer risk level function and all the age-dependent distributions of growth factor levels derived in the previous subsection, through an all-subsets regression using the Leap package (version 3.1) [101], which allowed for a comprehensive evaluation of the quality of our predictive model based on different combinations of the hormones/growth signals (Table S7).

### 4.9. Identification of Co-Expressed Clusters Among Cell Cycle Genes in Cancer

To identify co-expressed clusters among cell cycle genes across all samples of each cancer type, consensus clustering was applied to the expression matrix of core cell cycle genes using the ConsensusClusterPlus package (version 1.62.0) [102], which achieves high-quality clustering results through integrating multiple clustering solutions [103]. Specifically, its hierarchical clustering was performed 100 times on the expression matrix, using 80% of the core cell cycle genes in each iteration. The results from all 100 clustering iterations were combined to determine the final co-expressed gene clusters. Clusters containing fewer than 20 genes were excluded. The distance between two cell cycle genes were defined as follows:

(15) $$distance\left(i,j\right)=1-PCC\left(i,j\right)$$

where PCC(i,j) represents the Pearson correlation coefficient between gene i and gene j.

### 4.10. Regression Analyses for Each Co-Expressed Cluster of Cell Cycle Genes

To determine the activity of each co-expression cluster, we first performed principal component analysis (PCA) for each co-expression cluster for each cancer type. The first principal component (PC1) was extracted and used as a representative of the cluster. To simplify our discussion, we used the negative value of PC1 to represent the cluster if most of the genes in this cluster negatively correlated with PC1 (Figure S5).

To determine if the identified receptors and the level of nucleotide synthesis can explain the cell cycle progression, a regression analysis was conducted for PC1 of each cell cycle cluster against the expressions of the receptors for predicted growth signals and genes involved in de novo deoxyribonucleotides synthesis (Table S12), as well as PC1 of other cell cycle cluster(s), employing the forward selection procedure [104], which is a stepwise method that iteratively adds predictor variables to a model to maximize their contribution to explaining the response variable. The contribution by each receptor or deoxyribonucleotide synthesis to the regression model was evaluated using the Lindeman, Merenda, and Gold (LMG) method provided by the R package relaimpo (version 2.2.7) [105,106]. This method averages the contributions of each predictor to the R^2^ value across all possible orderings of the predictors.

To assess the generalization ability of our selected features, we conducted a random forest-based regression [107] of the PC1 of each cell cycle cluster against the selected features, using 10-fold cross-validation (CV). The R^2^ values for each cross-validation were then estimated. Additionally, four independent cancer RNA-seq datasets were obtained from GEO (Table S8). Only primary cancer samples were retained, and the expression data were transformed to the TPM-based measure. The PC1 for each cell cycle cluster was calculated using the same manner as described above. Each dataset was split into a training set and a test set covering 80% and 20% of the dataset, respectively. A random forest-based regression was employed to train the model, fitting PC1 of each cell cycle cluster against the selected features, and to test the model’s performance on the test set. R^2^ values for both the training and test sets were estimated.

### 4.11. Viral Infection Rate in the Cancer Tissue Samples and the Background Population

Data for cancer samples with viral infection, including hepatitis B (HBV), hepatitis C (HCV), and human papillomavirus (HPV), were retrieved from TCGA. We estimated that the amount of virus infecting a cancer tissue by mapping unmapped RNA-seq reads to the relevant viral sequences, following the idea of published studies [108,109].

To estimate the level of viral infection across the cancer tissue samples in a specific age interval, we calculated the infection level and the average age for each age interval of five years. If an age interval covered fewer than five cancer samples, the average infection rates from the neighboring intervals were used. Then, a regression analysis was conducted to find a continuous function that optimally fit the discrete age-specific infection rates for cancer patients aged between 20 and 90 (Table S9).

The infection data across the United States were collected from NHANES [100] and used as the background data. Individuals were classified as infected with HBV if they tested positive for both HBV surface antigen and HBV core antibody. For HCV infection, an active infection was determined by the presence of HCV RNA. We also collected data on HPV infection. A regression analysis was conducted for cancer patients to estimate the age-dependent viral infection rates across the general US population, as detailed in Table S9.

### 4.12. Pathway-Based Analyses of Gender Differences in Normal Tissues

To investigate the biological differences between male and female populations, we began by identifying DEGs between the two populations in GTEx, defined as having a fold change > 1.5 and FDR < 0.05, using DESeq2. Tissue types with fewer than 10 samples from either gender were excluded from our study to ensure the statistical reliability. We then conducted pathway and functional category enrichment analyses among the DEGs.

### 4.13. Estimating Iron Accumulation in Blood

To estimate iron accumulation in the blood, we collected data on serum iron, ferritin concentration, transferrin saturation, and hemoglobin concentration from the NHANES dataset [100]. Differences between males and females were calculated using the Wilcoxon test. Differences were considered statistically significant at a *p*-value < 0.05.

### 4.14. Perturbation Analysis

To investigate potential relationships between the blood ferritin level and the occurrence rates of different cancer types, we used the NHANES data [100] for the perturbation analysis in each cancer types as follows: for a given set of n cancer samples and m non-cancerous control samples, we randomly reassigned the n+m labels to the samples so each sample had a distinct label. We repeated this for 100,000 iterations. For each iteration, we calculated the average ${ferritin}_{i}$ in the samples assigned with the cancer label. The *p*-value was then estimated as follows:

(16) $$sign(x)=\left\{\begin{array}{ll} 1, & x>{ferritin}_{cancer}\\ 0, & x\leq {ferritin}_{cancer} \end{array}\right.$$

(17) $$Pvalue=\frac{\sum_{i=1}^{100,000}sign\left({ferritin}_{i}\right)}{100,000}$$

where ${ferritin}_{cancer}$ is the average ferritin level across the actual cancer samples. The cutoff for statistical significance is a *p*-value < 0.05.

### 4.15. Quantitative Analyses of Different Age-Dependent Cancer Occurrence Rates Between Genders

To quantitatively analyze the differences in age-dependent cancer occurrence rates between males and females, we estimated this variable at each ${age}_{i}$ as follows:

(18) $$f_{d}({age}_{i})={log}_{2}(\frac{f_{male}({age}_{i})}{f_{female}({age}_{i})})$$

where $f_{male}({age}_{i})$ and $f_{female}({age}_{i})$ represent the cancer occurrence rates at ${age}_{i}$ for males and females, respectively. We used the ratio here rather than the absolute differences, because cancer occurrence rates can vary significantly across different ages. For cancer types with a higher occurrence rate in males, the iron levels in the corresponding normal tissues were used to study variations in occurrence rates. Specifically, a regression analysis was conducted for the differences in occurrence rates against the fold changes (FC) in the expression of iron genes between males and females, where the fold change in the iron gene j was calculated for each ${age}_{i}$ as follows:

(19) $${FC}_{j}({age}_{i})={log}_{2}(\frac{{median(exp}_{j\;male}({age}_{i}))+1}{{median(exp}_{j\;female}({age}_{i}))+1})$$

where ${exp}_{j\;male}({age}_{i})$ and ${exp}_{j\;female}({age}_{i})$ represent the expressions of iron-related gene j in GTEx at ${age}_{i}$ for males and females, respectively. The sets of iron-related genes are detailed in Table S12. Forward selection was employed to identify the optimal combination of factors to explain the variations in occurrence rates between genders.

For thyroid cancer, which has a higher occurrence rate in females, the FC of the circulation *E2* was analyzed to study the variations in occurrence rates.

### 4.16. Functional Analyses of Cancer Types with Early vs. Late Peak Ages

To investigate the differences between cancer types with early vs. late occurrence peak ages, we classified the cancer types under study into two groups: those with early peak ages (peak age < 75 years) and those with late peak ages (peak age ≥ 75 years). RNA-seq data from TCGA were grouped accordingly, and differential expression analysis was conducted using DESeq2. The differentially expressed genes (DEGs), ordered by fold change, served as inputs for the gene set enrichment analysis (GSEA) [110]. This analysis was employed to identify statistically significant differences in pathway activities, specifically within Gene Ontology Biological Processes (GO BP), between the two groups. Enriched pathways having |NES| > 1 with FDR < 0.05 were considered statistically significant, where NES is a normalized enrichment score. Table S11 lists the GO BPs with significant differences between the two groups.

## 5. Conclusions

In this study, we analyzed the age-dependent occurrence rate distributions of 23 cancer types. Our finding revealed two major contributors to each unimodular distribution: the age-dependent risk for cancer development in the relevant organ and the availability level of bloodborne growth signals specifically required by the relevant cancer. In addition, we also offered a reason for the additional peak in each of the two bimodular distributions by cervical and liver cancers, namely viral infection. Further analyses of these modeling results strongly suggest the possibility to treat cancer through cleaning up the growth signals in the blood circulation, specifically required by a cancer, offering a fundamentally novel and general strategy for treating cancers.

In conclusion, this study provides valuable insights into the multifactorial nature of age-dependent cancer occurrence rate, with implications for cancer prevention, early detection, and therapeutic strategies. Further research is needed to explore the molecular mechanisms linking these factors, as well as the role of genetic predispositions, environmental exposures, and lifestyle factors in shaping cancer risk across the lifespan.

## Figures and Tables

**Table 1:** Mapping between SEER cancer sites and TCGA cancer types.

TCGA Cancer Type	Cancer Site in SEER
Bladder urothelial carcinoma (BLCA)	Urinary bladder
Breast invasive carcinoma (BRCA)	Breast
Cervical squamous cell carcinoma and endocervical adenocarcinoma (CESC)	Cervix uteri
Cholangiocarcinoma (CHOL)	Intrahepatic bile duct
Colon adenocarcinoma (COAD)	Colon excluding rectum
Esophageal carcinoma (ESCA)	Esophagus
Glioblastoma (GBM)	Glioblastoma
Kidney chromophobe (KICH)	Kidney
Kidney renal clear cell carcinoma (KIRC)	Kidney
Kidney renal papillary cell carcinoma (KIRP)	Kidney
Liver hepatocellular carcinoma (LIHC)	Liver
Lung adenocarcinoma (LUAD)	Adenocarcinoma of the lung and bronchus
Lung squamous cell carcinoma (LUSC)	Squamous cell carcinoma of the lung and bronchus
Mesothelioma (MESO)	Mesothelioma
Ovarian serous cystadenocarcinoma (OV)	Ovary
Pancreatic adenocarcinoma (PAAD)	Pancreas
Prostate adenocarcinoma (PRAD)	Prostate
Rectum adenocarcinoma (READ)	Rectum
Skin cutaneous melanoma (SKCM)	Melanoma of skin
Stomach adenocarcinoma (STAD)	Stomach
Testicular germ cell tumors (TGCT)	Testis
Thyroid carcinoma (THCA)	Thyroid
Uterine corpus endometrial carcinoma (UCEC)	Corpus uteri

**Table 2:** The accuracy levels and predicted growth signals for regression models of seven cancer types.

Cancer	Accuracy Level of the Model $(R_{adjust}^{2}$)	Predicted Growth Signals	References in Support of the Prediction
ESCA	0.9979	*BMP6*, *TGFα*, *TGFβ1*	[16,17,18,19,20,21,22,23]
TGCT	0.9956	*BMP2*, *INHBC*	[24,25,26,27,28]
THCA	0.9905	*E2*, *TGFβ1*	[29,30,31]
COAD	0.9955	*BTC*	[33]
LUAD	0.9784	*BMP6*, *BTC*	[34,35]
PAAD	0.9996	*PDGFD*, *TGFβ1*	[36,37,38]
SKCM	0.9884	*TGFβ1*	[39]

**Table 3:** Mapping between cancer types and GTEx tissue types.

Cancer	Tissue Type in GTEx	#Samples in GTEx	#Male Samples in GTEx	#Female Samples in GTEx	Cancers with Gender Difference
BLCA	Bladder	21	14	7	-
BRCA	Breast	168	-	168	-
CESC	Cervix uteri	19	-	19	-
CHOL	-	-	-	-	-
COAD, READ	Colon	779	499	280	√
ESCA	Esophagus	555	363	192	√
GBM	Brain	464	334	130	√
KICH, KIRC, KIRP	Kidney	85	66	19	√
LIHC	Liver	226	161	65	√
LUAD, LUSC	Lung	578	395	183	√
MESO	-	-	-	-	-
OV	Ovary	180	-	180	-
PAAD	Pancreas	328	207	121	√
PRAD	Prostate	245	245	-	-
SKCM	Skin	1305	878	427	√
STAD	Stomach	359	227	132	√
TGCT	Testis	361	361	-	-
THCA	Thyroid	653	434	219	√
UCEC	Uterus	142	-	142	-

## Data Availability

All data utilized in this study are derived from publicly available datasets. Detailed information regarding these data sources, including specific dataset identifiers and access links where applicable, is provided within the article.

## Supplementary Materials

The following supporting information can be downloaded at: https://www.mdpi.com/article/10.3390/ijms26010275/s1.
