# Supplementary material for: Elucidation of Factors Affecting the Age-Dependent Cancer Occurrence Rates
Source: Int J Mol Sci. 2024 Dec 31;26(1):275. doi: 10.3390/ijms26010275 (PMC11720044; doi:10.3390/ijms26010275)
Supplement: Supplementary file 1 [file ijms-26-00275-s001.zip › supplementary figure.pdf]

SUPPLEMENTARY FIGURES AND CPATIONS

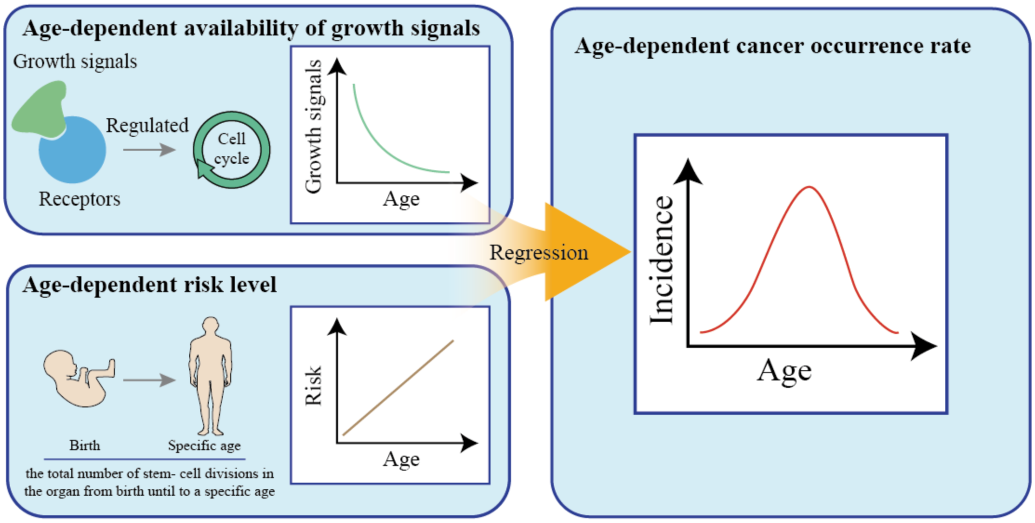

Figure S1. Schematic illustration of the regression model for age-dependent cancer occurrence rate. The model consists of two factors: the availability levels of specifically needed growth signals and the age-dependent risk level.

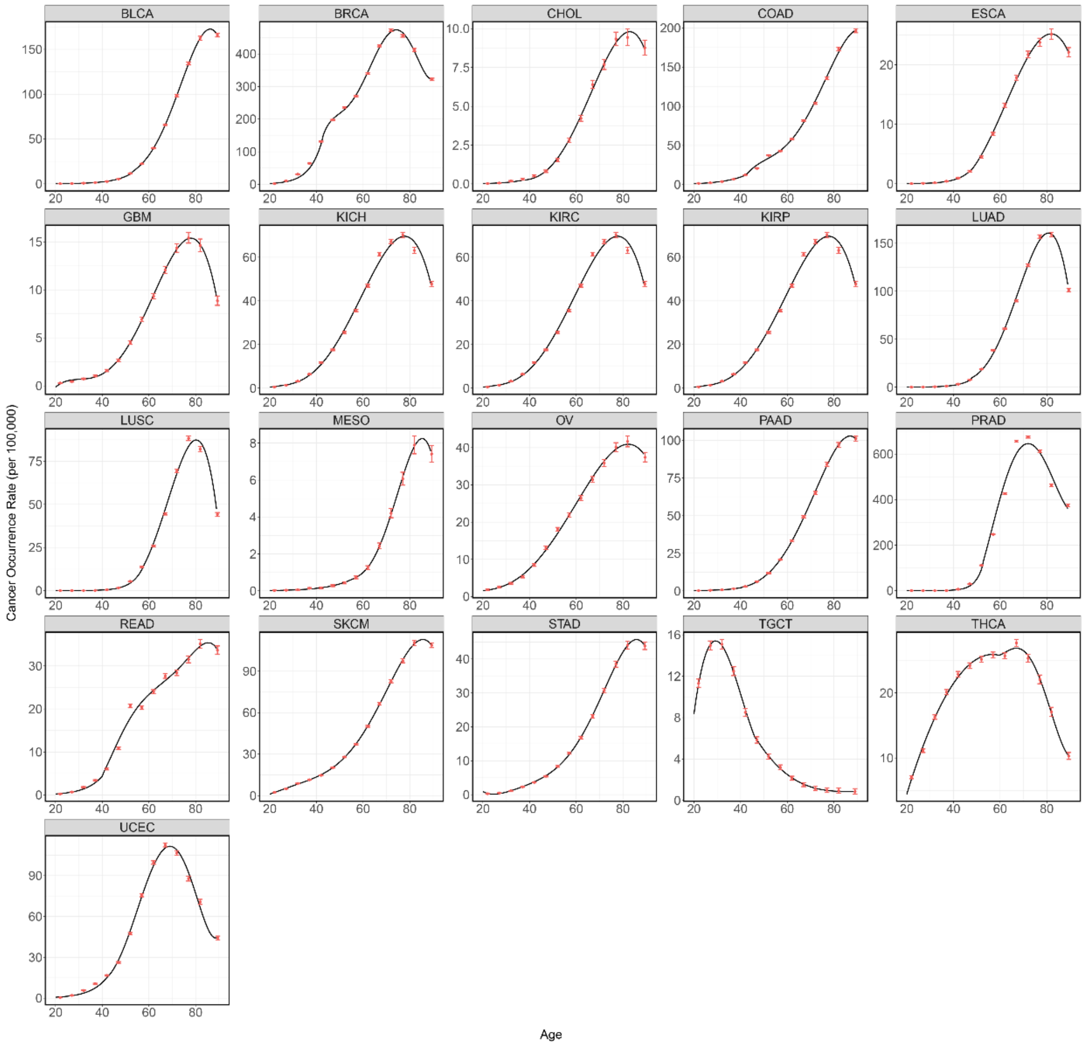

Figure S2. Regression models fitting the cancer occurrence rates with unimodal distributions. The 95% confidence intervals are depicted using red lines.

(1) Higher Cancer Occurrence Rates in Males Compared to Females

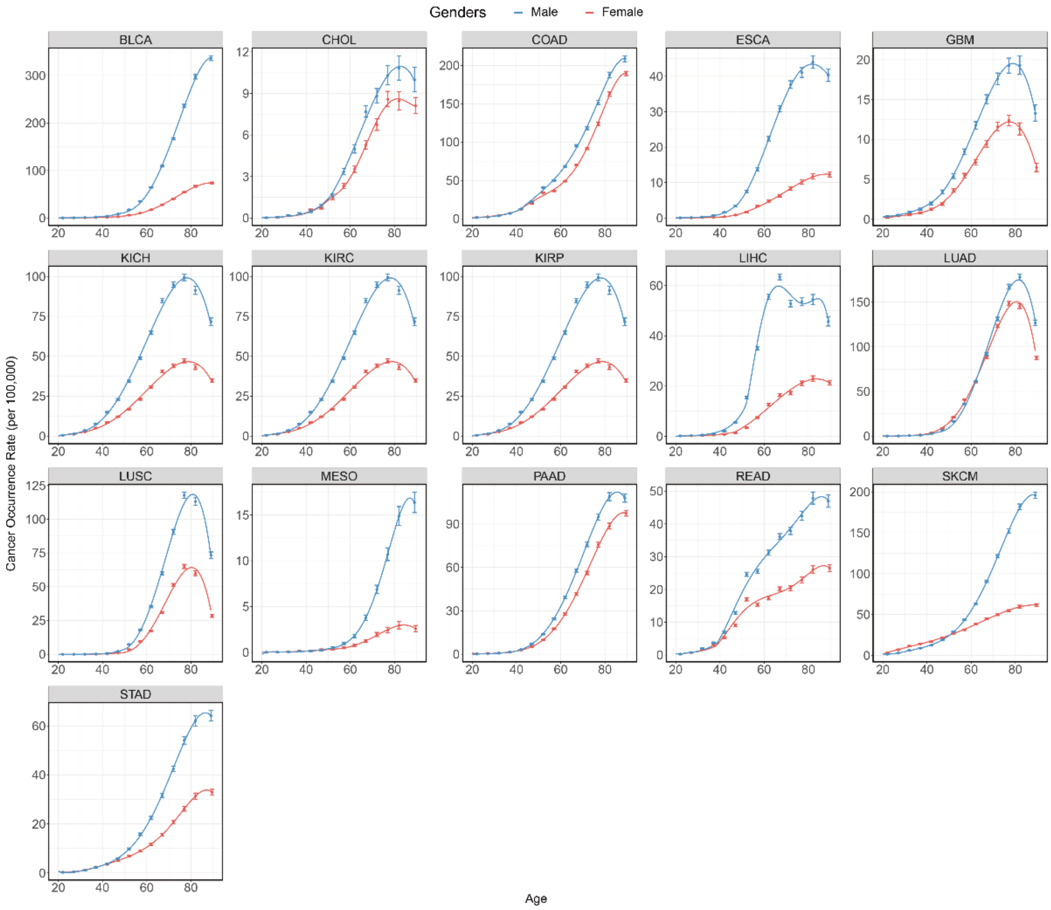

(2) Higher Cancer Occurrence Rates in Females Compared to Males

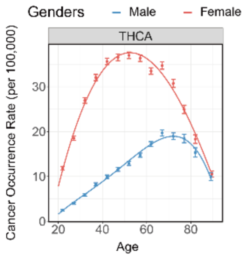

Figure S3. Regression models fitting cancer occurrence rates for different cancers in male and female, respectively. The 95% confidence intervals are depicted by red lines.

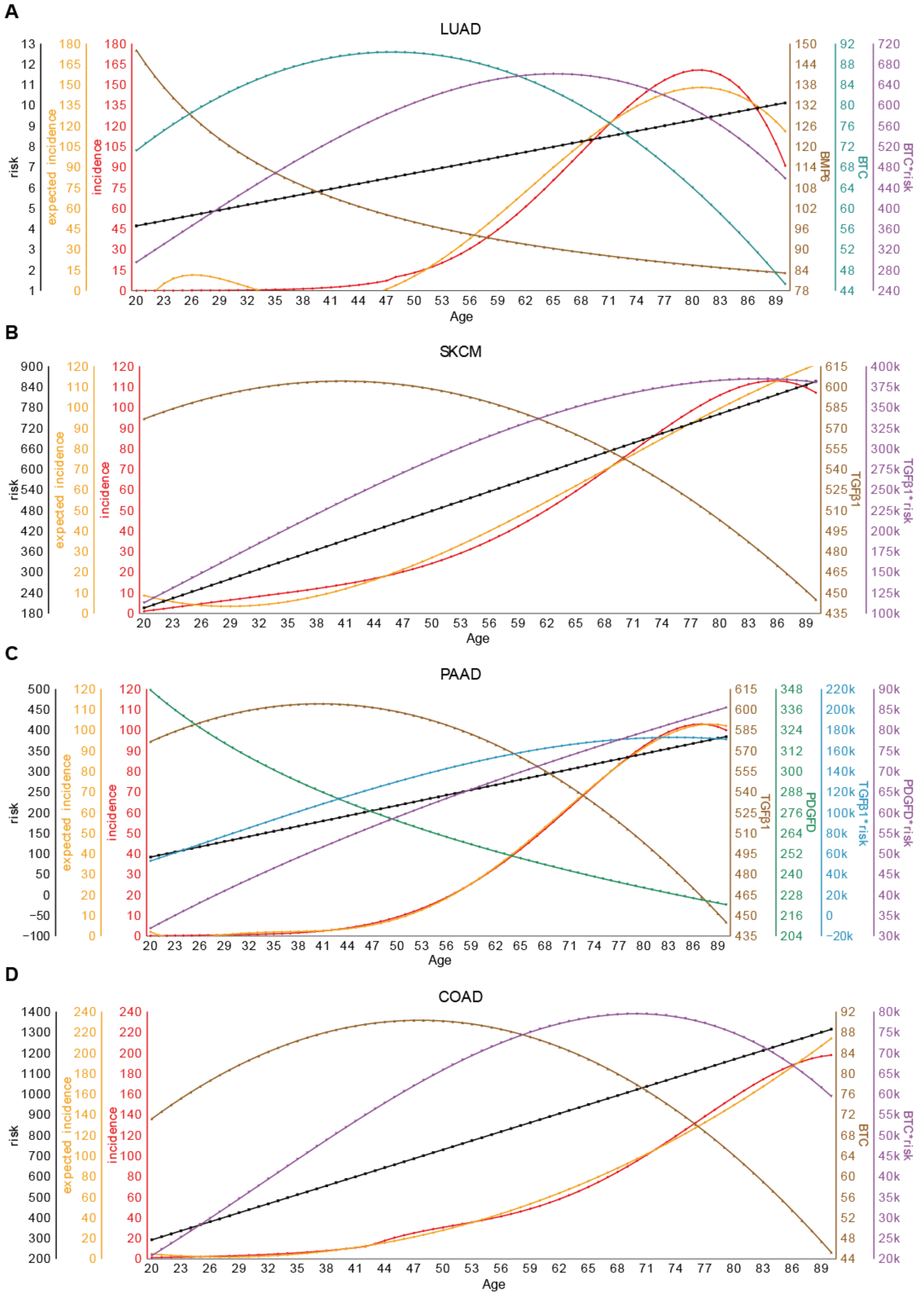

Figure S4. Regression models fitting age-dependent (x-axis) cancer occurrence rates against cancer risk and the availability levels of specific growth signals. (A-D) The regression models for (A) LUAD, (B) SKCM, (C) PAAD and (D) COAD, respectively. In each panel, the red line is for the cancer occurrence rate; the orange line for the predicted cancer occurrence rate by the regression model; the black line for the cancer risk, and the other lines for age-dependent levels of circulatory growth signals. * in the figure denotes the interaction term between growth signals and cancer risk.

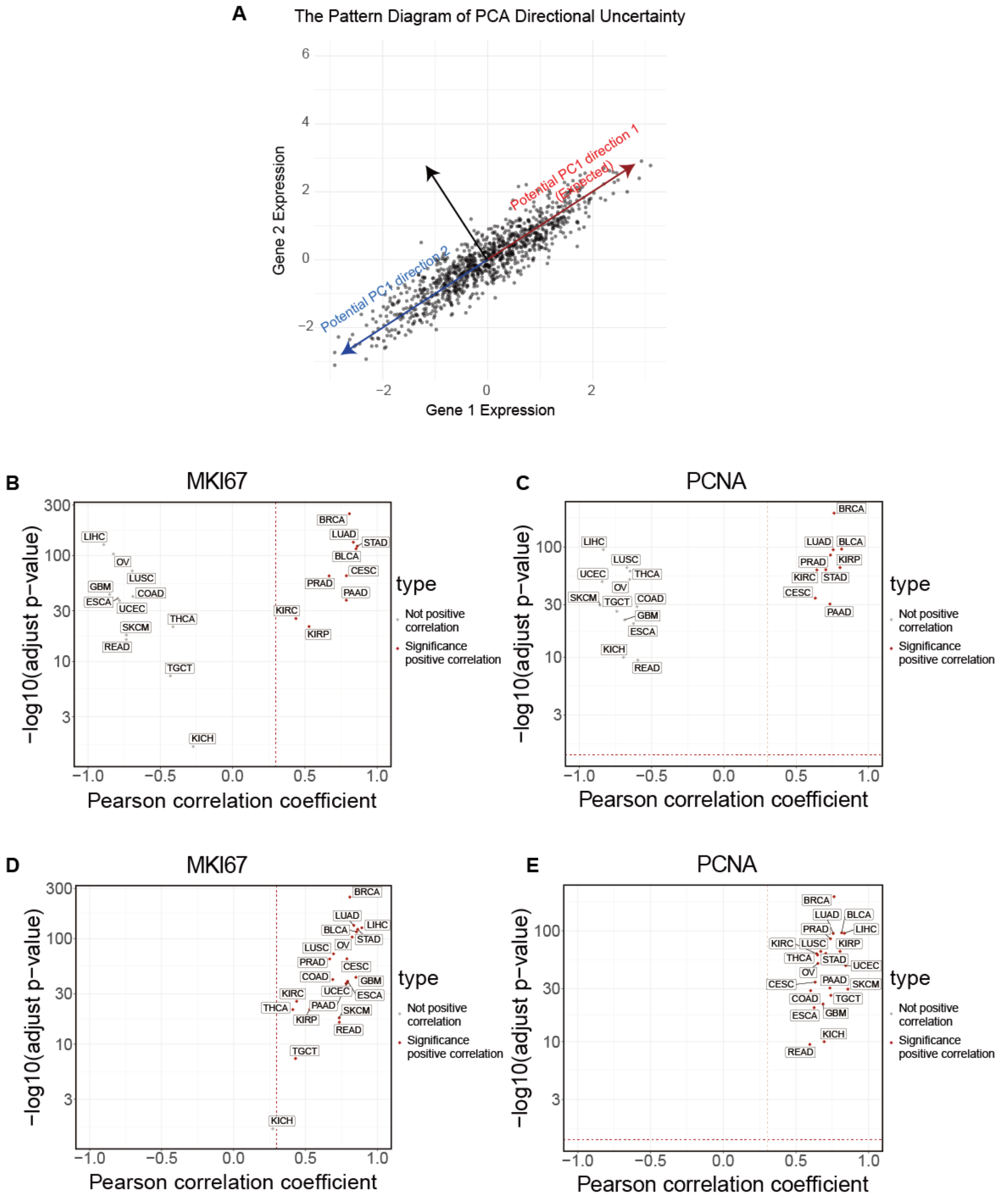

Figure S5. The principal components analysis (PCA) for cell cycle genes. (A) Pattern diagram illustrating multiple potential directions for PC1 in PCA, where the anticipated direction is positively correlated with most cell cycle genes. (B-C) Pearson correlation coefficients between unadjusted PC1 of core cell cycle genes and two genes associated with cancerous cell proliferation: MKI67 (B) and PCNA (C). (D-E) Pearson correlation coefficients for adjusted PC1 of core cell cycle genes with the same proliferation-related genes: MKI67 (D)

and PCNA (E). The vertical red dotted line in (B-E) indicates a p-value of 0.01, while the horizontal one signifies a rho value of 0.3. Most cancers demonstrate a significant positive correlation between adjusted PC1 for core cell cycle genes and these proliferation markers, highlighting the efficacy of the adjustment.

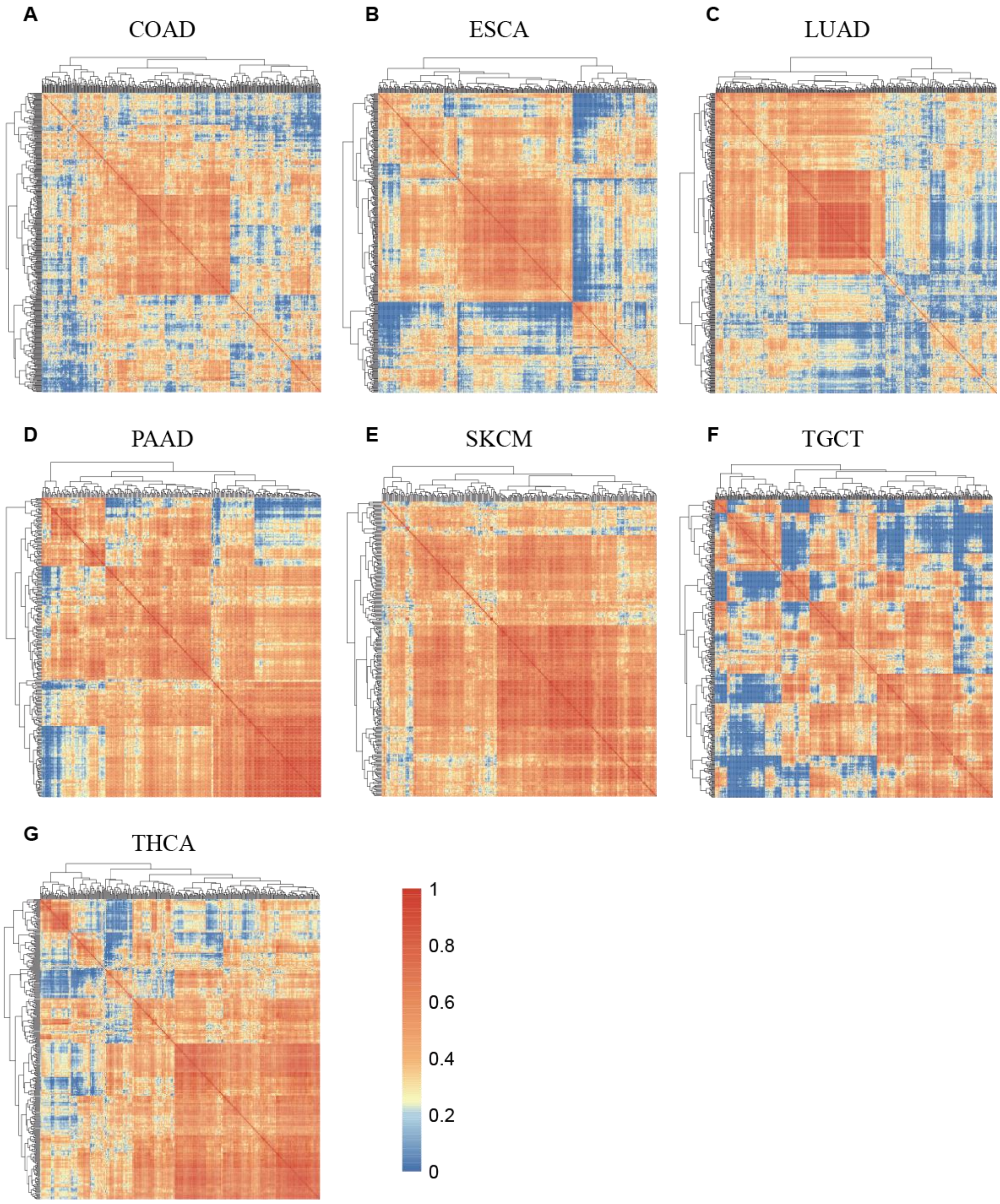

Figure S6. The heatmap of the co-expression for core cell-cycle genes in 7 cancer types.

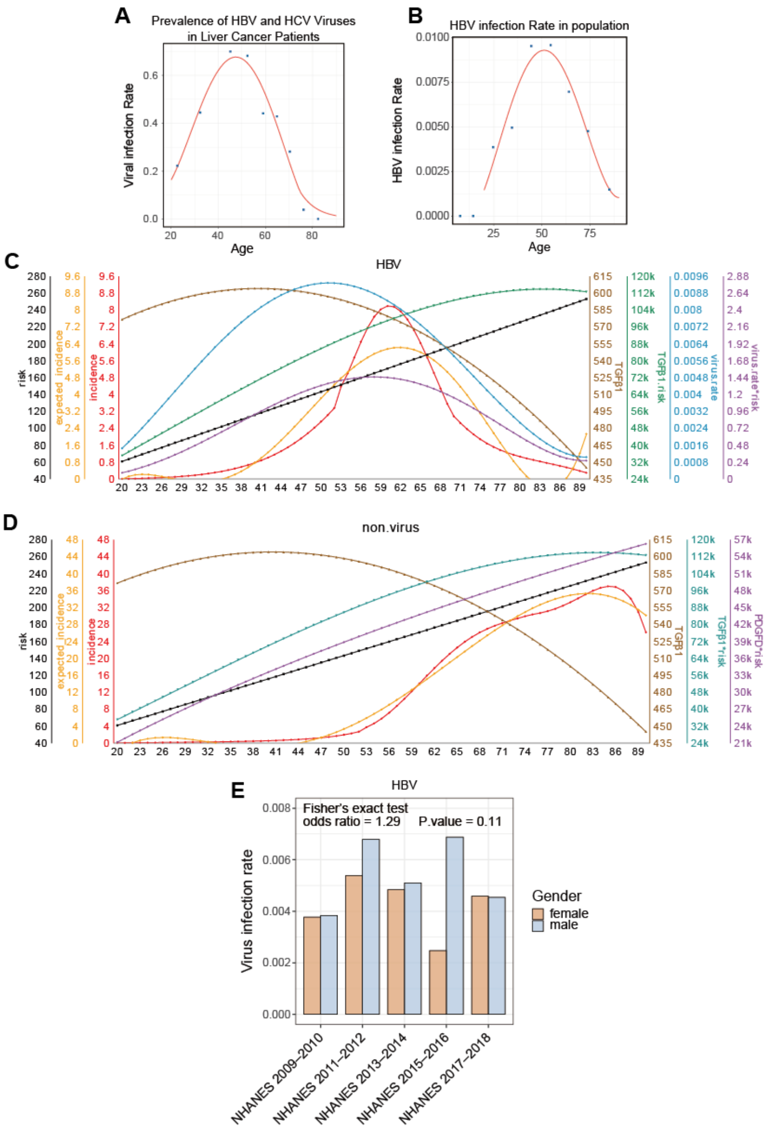

Figure S7. HBV and HCV infection rates in liver cancer. **(**A) A regression model for age-dependent HBV or HCV infection rates in liver cancer patients. **(**B) A regression model for HBV infection rates in the population of USA. (C) A regression models for age-dependent liver cancer occurrence rates with HBV infection against age-dependent cancer risk levels, age-dependent HBV infection rates in the population of the USA and the levels of growth signals needed by liver cancers. (D) A regression model for age-dependent cancer occurrence rates without viral infections against age-dependent cancer risk levels and the levels of growth signals in circulation. (E) HBV infection rates across male, female at different age groups in the USA.

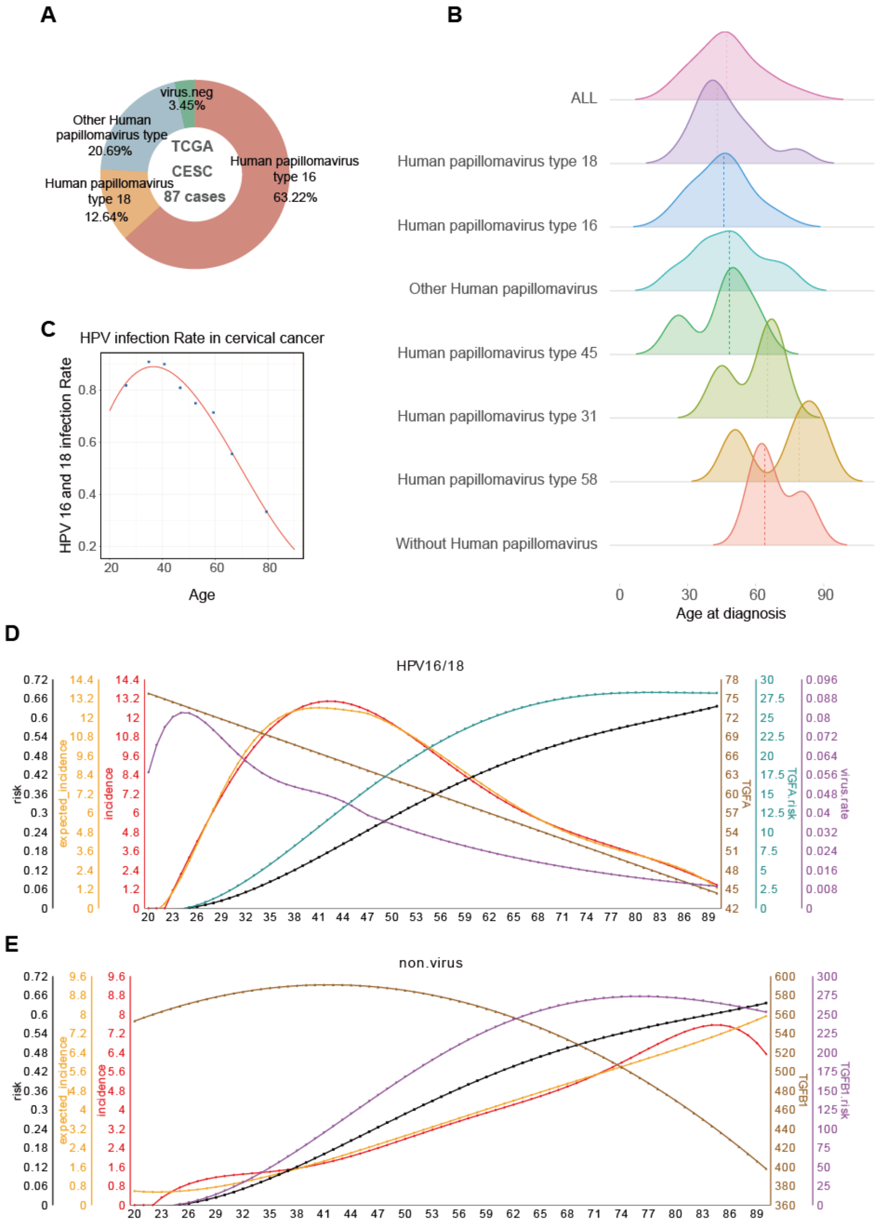

Figure S8. HPV infection rates in cervical cancer. (A) Infection rates of multiple HPV types in cervical cancer. (B) The age distribution of cervical cancer patients with infection by different HPV types. (C) A regression model of combined infection rates by HPV 16 and 18 in cervical cancer. (D) A regression models for age-dependent cervical cancer occurrence rates with HPV 16/18 infection against age-dependent cancer risk levels, age-dependent HPV 16/18 infection rates in the population of the USA and the levels of growth signals needed by cervical cancer. (E) A regression model for age-dependent cancer occurrence rates without HPV 16/18 infections against age-dependent cancer risk levels and the levels of growth signals in circulation.

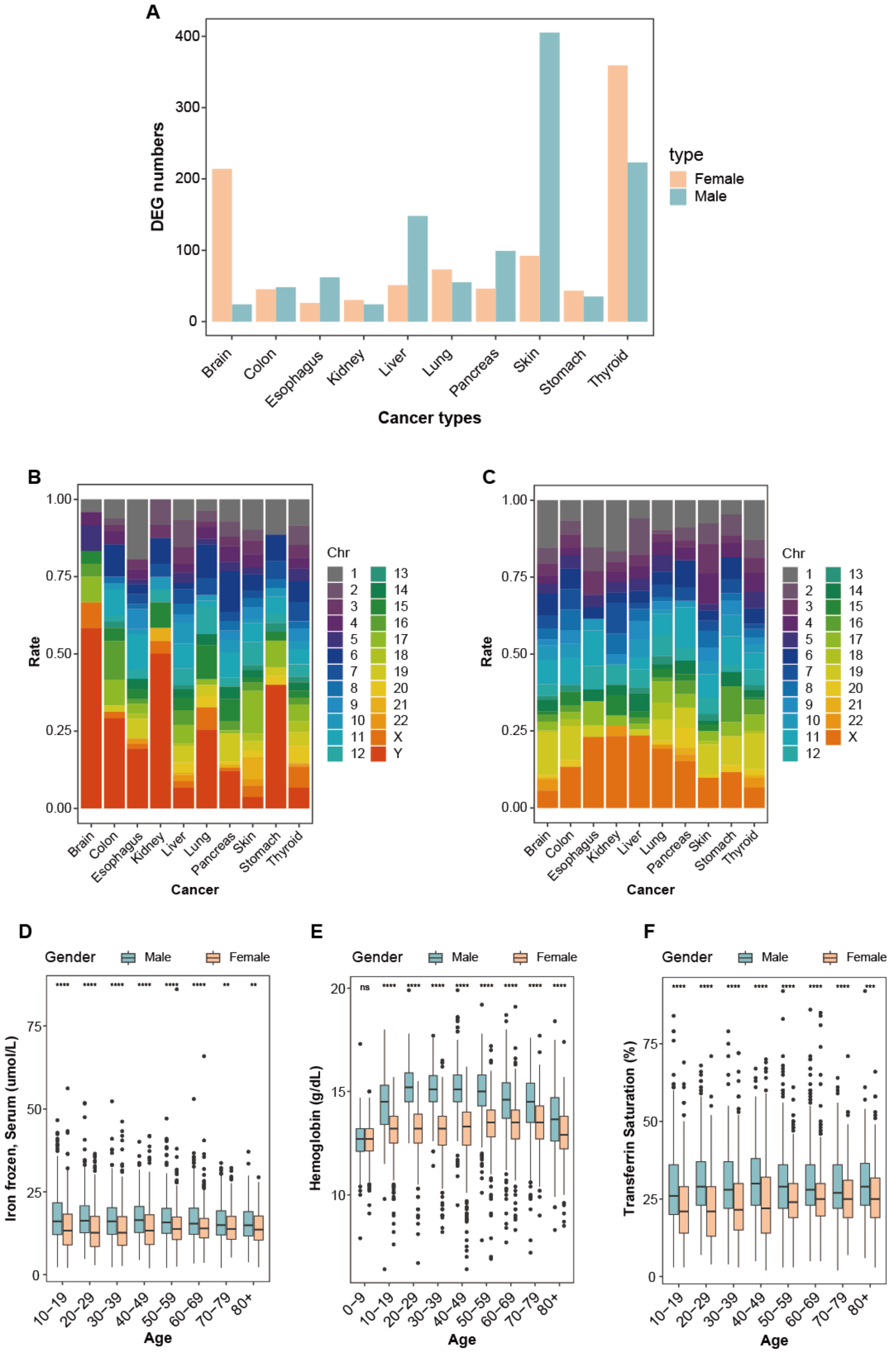

Figure S9. Gender-specific gene expression and differential iron levels across various tissues. (A) Numbers of genes differentially expressed between males and females across different tissues. (B-C) Numbers of upregulated genes across different chromosomes for each tissue type in males (B) and female (C), respectively. (D-F) Box plots for blood iron levels (D), blood-hemoglobin levels (E) and transferrin levels (F) in male vs. female across various age groups, respectively. Significance levels are defined as follows: 'ns' for not significant, * for p-value ≤ 0.05, '**' for p-value ≤ 0.01, '***' for p-value ≤ 0.001, and '****' for p-value ≤ 0.0001.

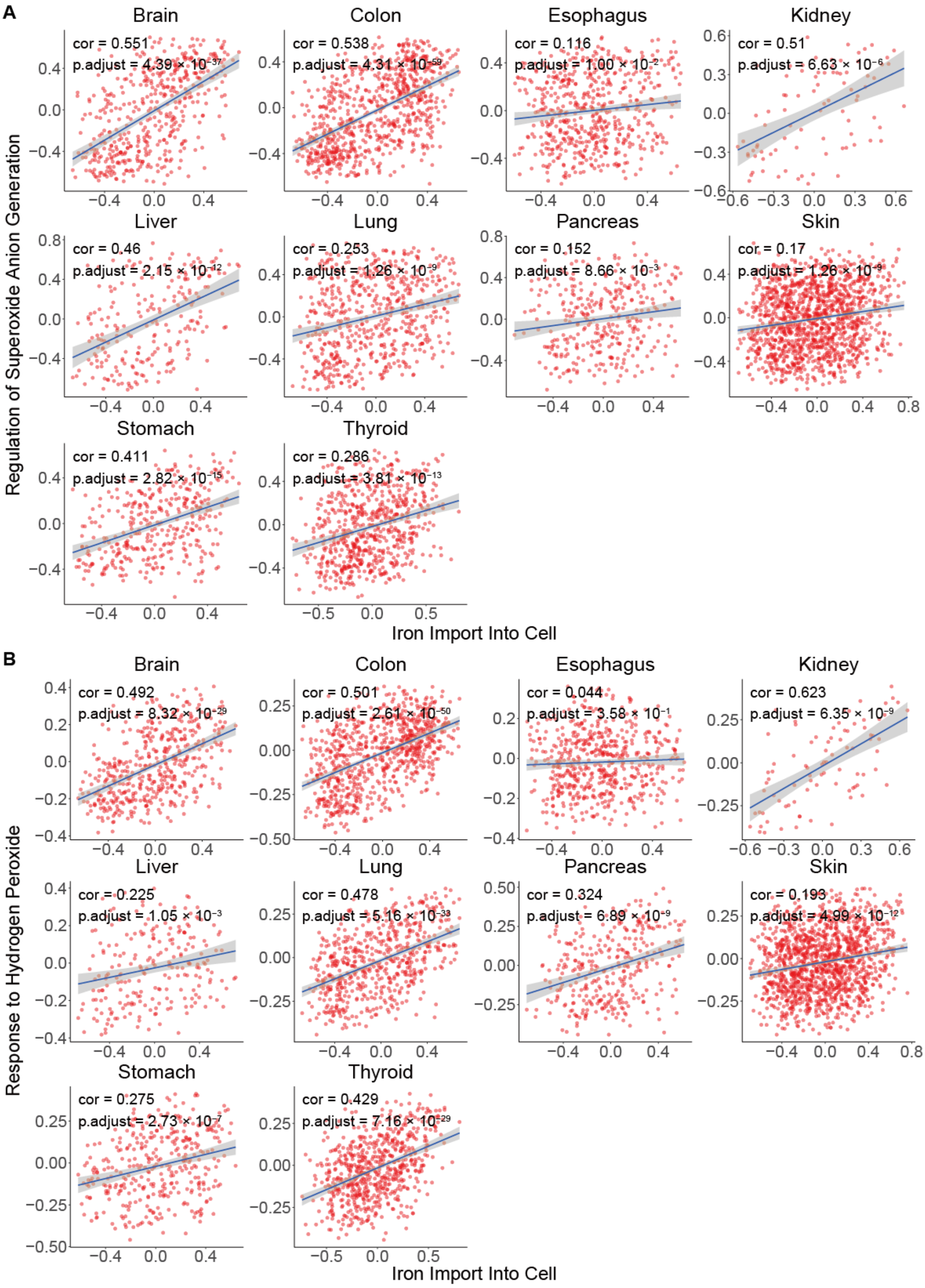

Figure S10. Scatter plots for tissue samples of different organs, each represented by the iron level and the oxidative stress derived from gene-set analyses.

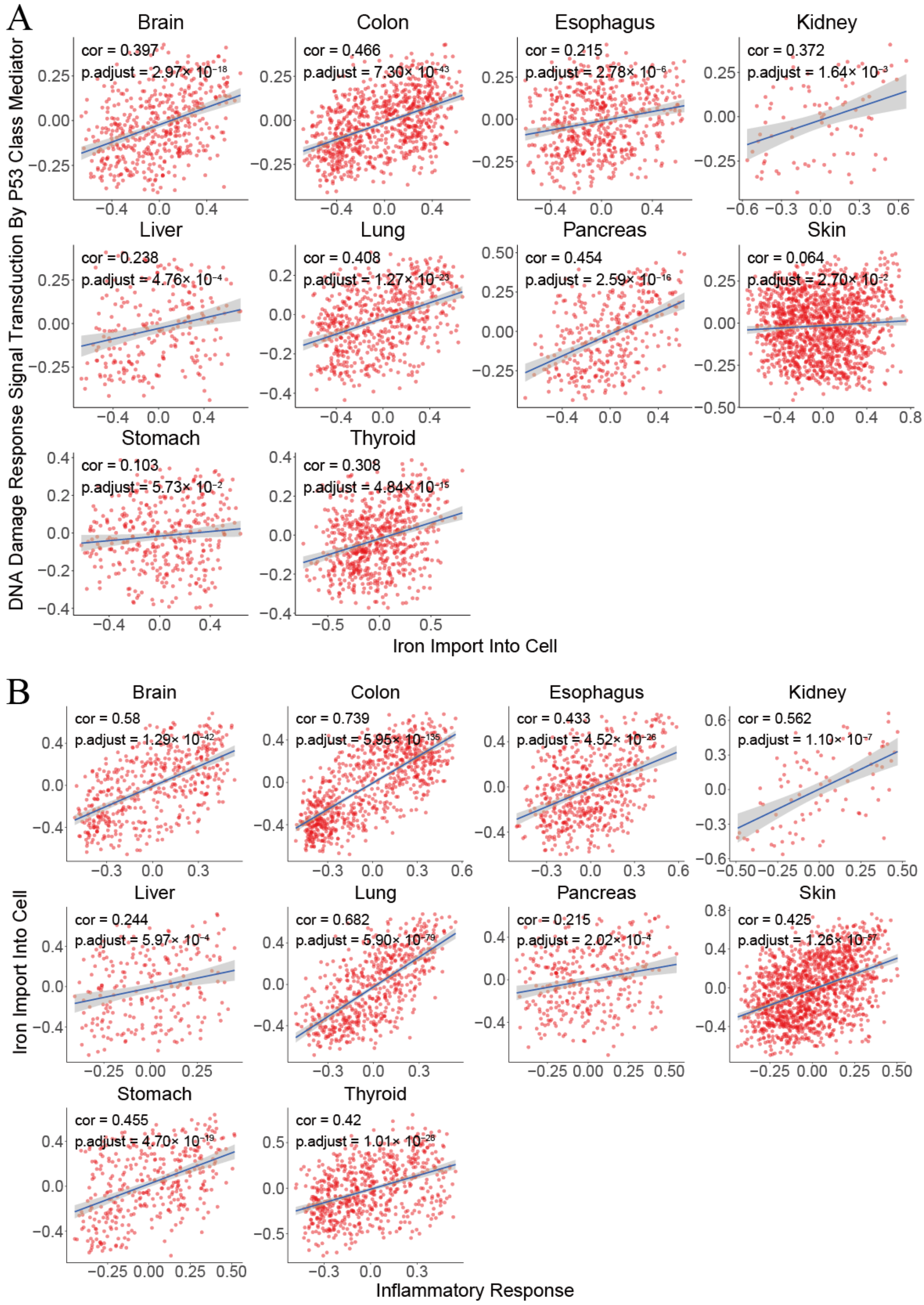

Figure S11. Scatter plots for tissue samples of different organs, each represented by the iron level and DNA damage level and the level of inflammation.

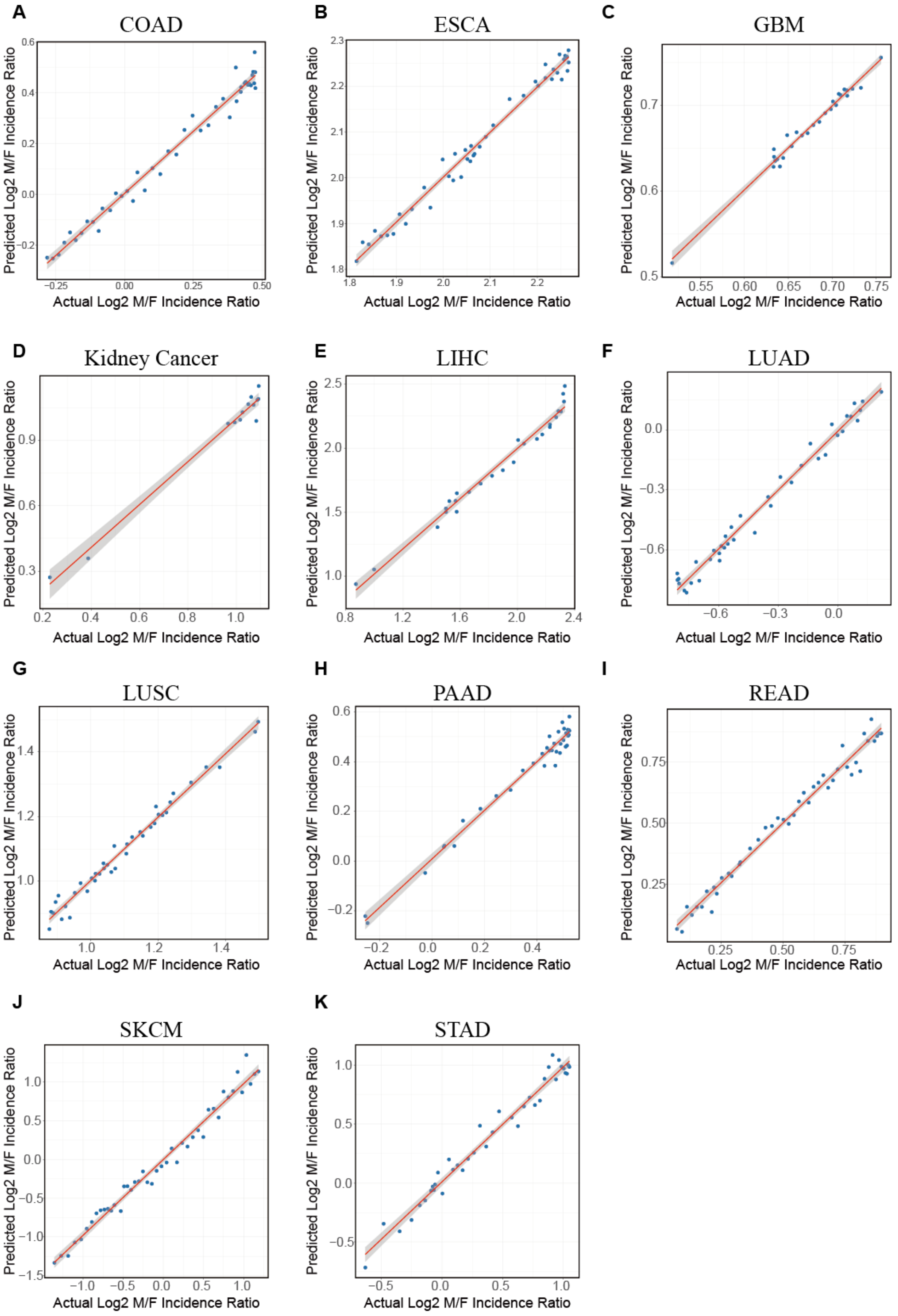

Figure S12. Scatter plots comparing the actual cancer incidence ratio (male vs. female) in different ages with those predicted by expression difference of iron-related genes for different cancer types, illustrating the consistency of these predictions.

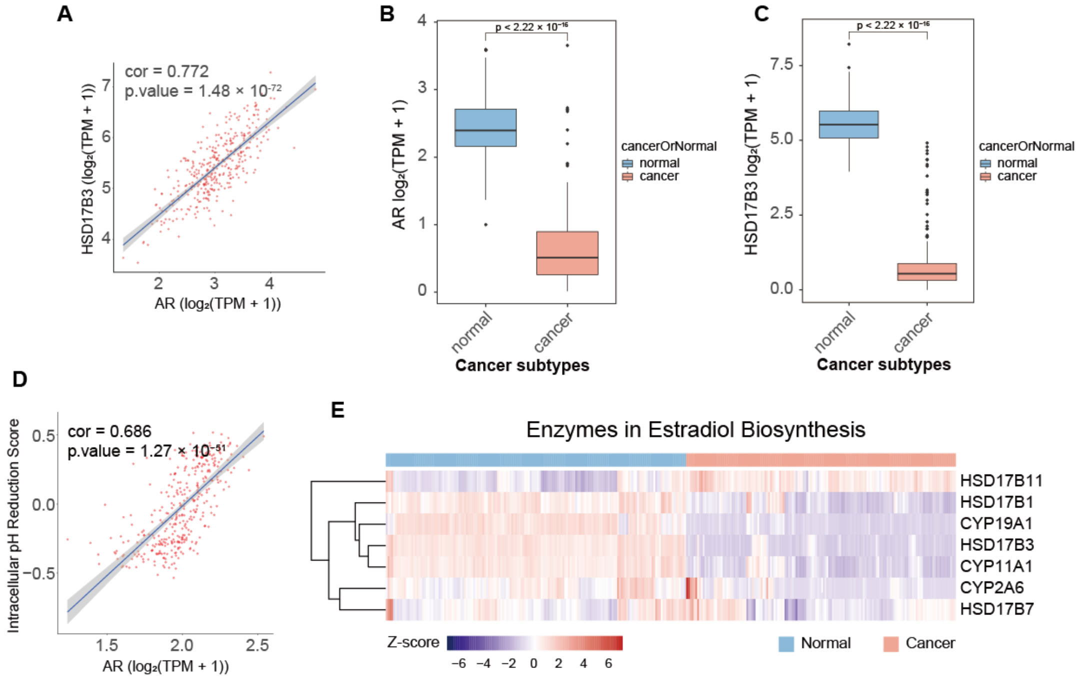

Figure S13. The enzymes and receptors for sex hormones in normal and cancerous testicular tissues. (A) Scatter plot showing the correlation between *HSD17B3* and *AR* in normal testicular tissue. (B-C) Box plots for differential expressions of *HSD17B3* and *AR* between normal and cancerous testicular tissues, respectively. (D) Correlations between *AR* expression and intracellular pH reduction signals in normal testicular tissue. (E) Heatmap displaying the differential expression of enzyme genes involved in estradiol biosynthesis between normal tissues and cancerous tissues.

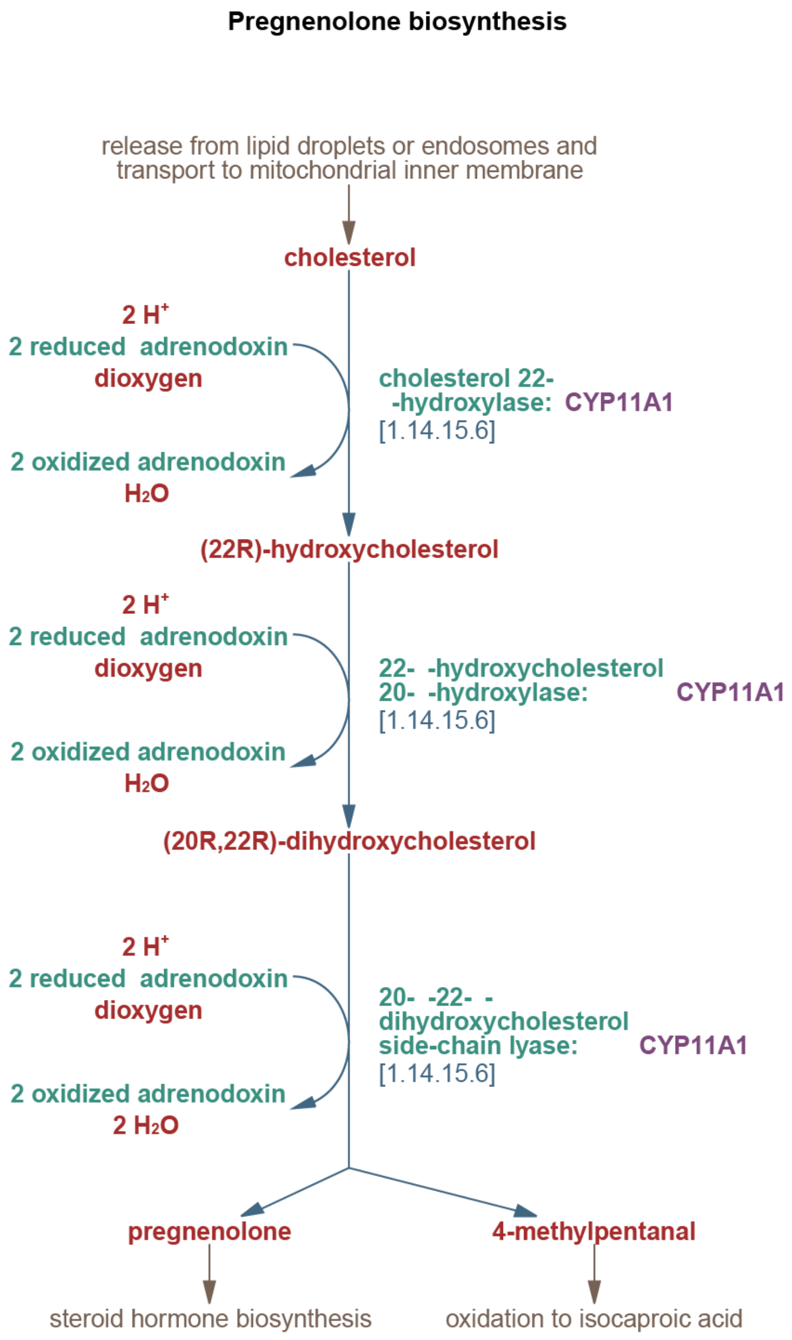

Figure S14. Pregnenolone biosynthesis pathway from HumanCyc database.

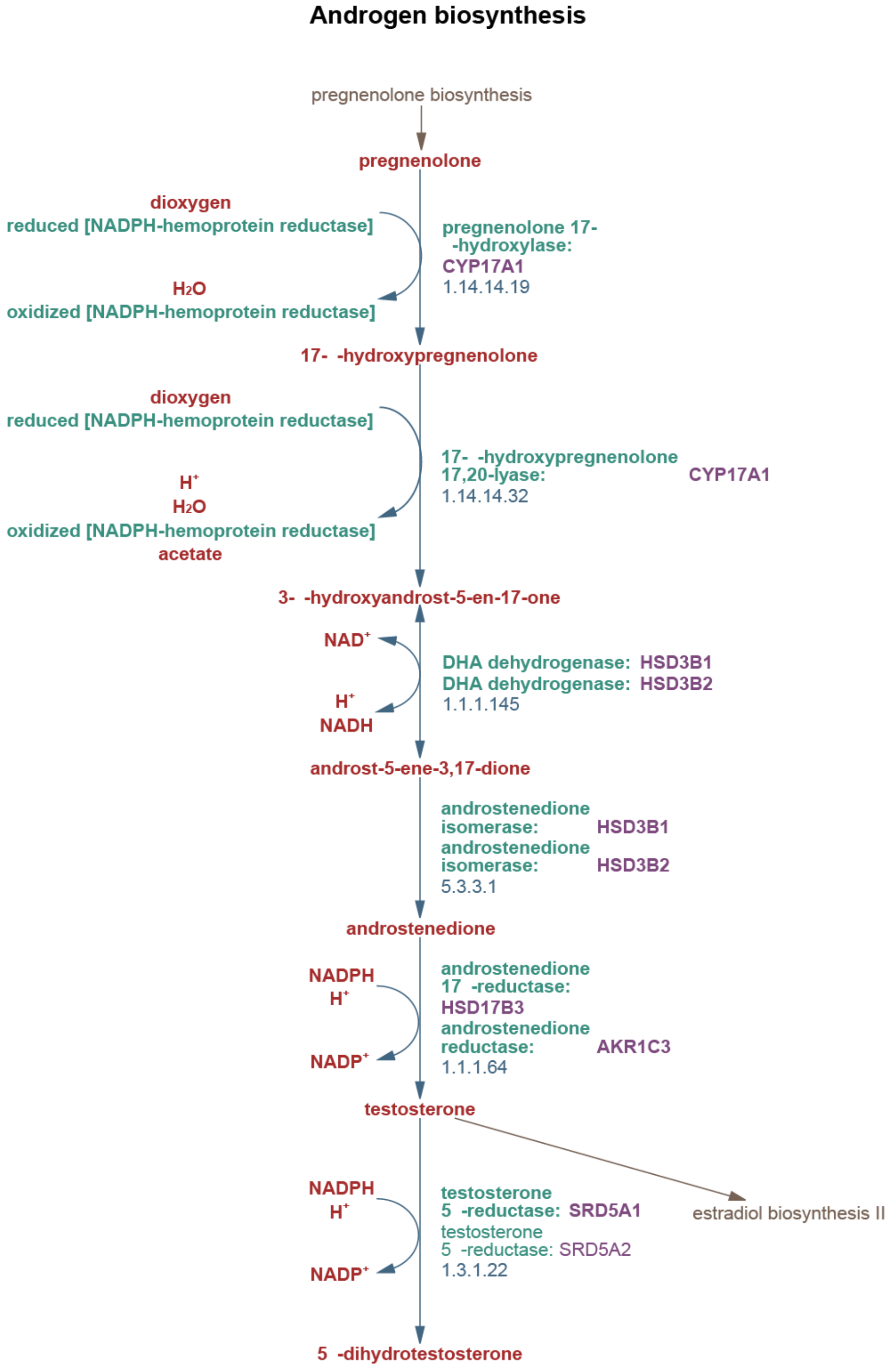

Figure S15. Androgen biosynthesis pathway from HumanCyc database.

Estradiol biosynthesis I (via estrone)

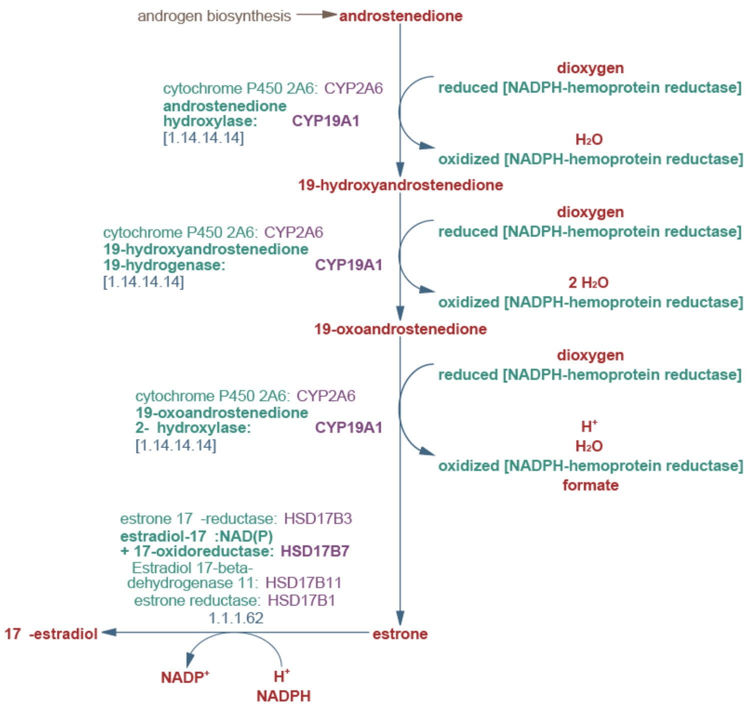

Estradiol biosynthesis II

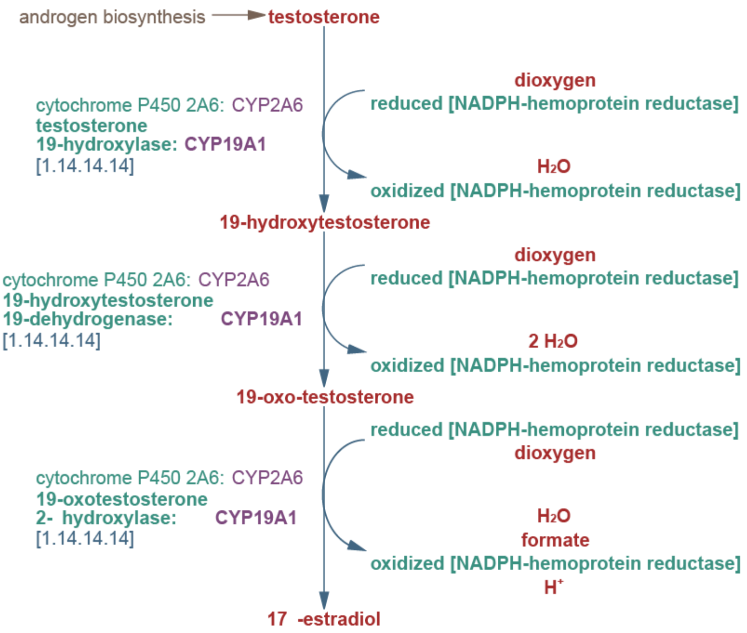

Figure S16. Estradiol biosynthesis pathway from HumanCyc database.

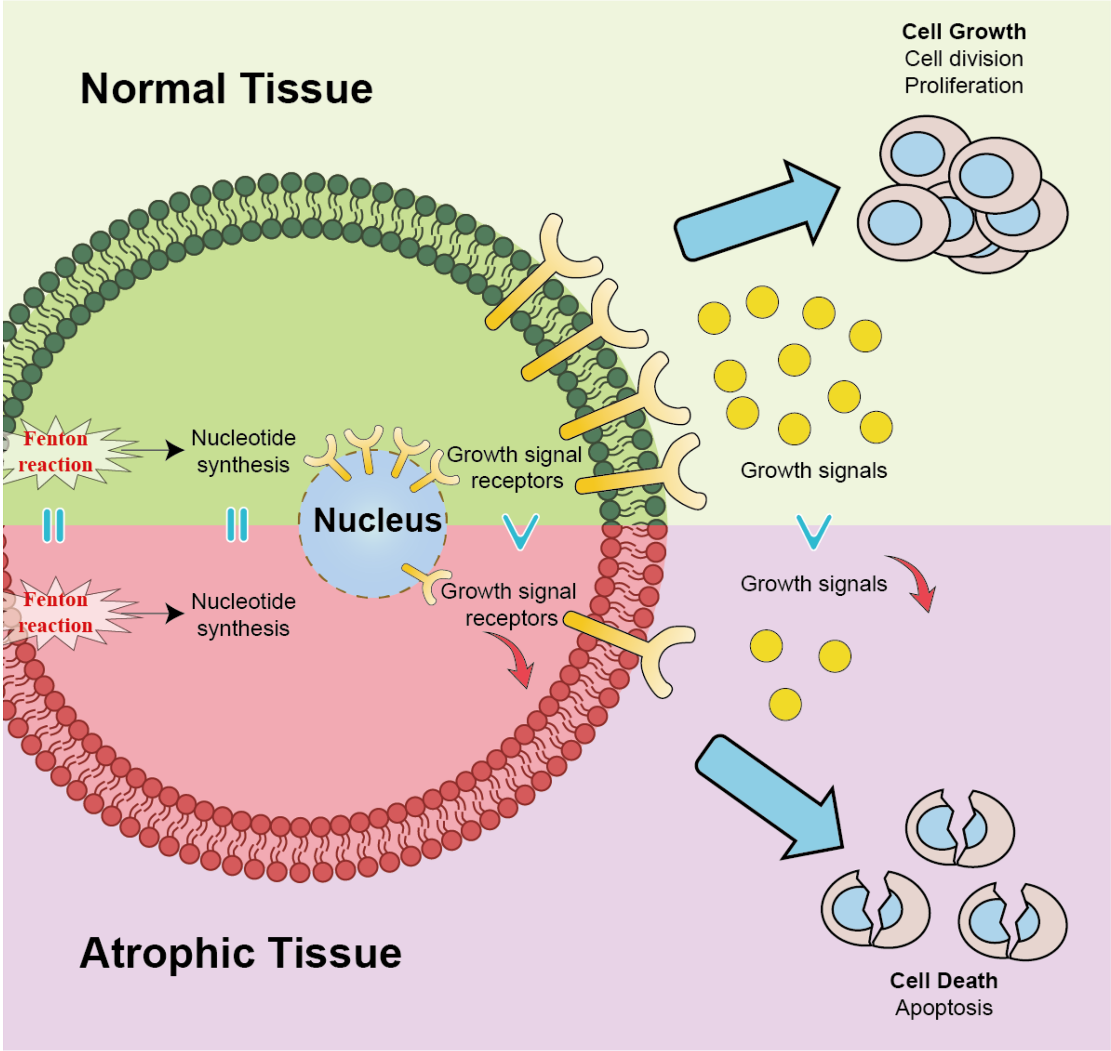

Figure S17. Model for cells death in the form of tissue atrophy. This model illustrates how tissue atrophy results from an imbalance between nucleotide synthesis, influenced by the Fenton reaction, and growth signals mediated by receptors.
